# Macrophages enhance Vegfa-driven angiogenesis in an embryonic zebrafish tumour xenograft model

**DOI:** 10.1242/dmm.035998

**Published:** 2018-11-29

**Authors:** Denver D. Britto, Barbara Wyroba, Wenxuan Chen, Rhoswen A. Lockwood, Khanh B. Tran, Peter R. Shepherd, Christopher J. Hall, Kathryn E. Crosier, Philip S. Crosier, Jonathan W. Astin

**Affiliations:** 1 Department of Molecular Medicine and Pathology, School of Medical Sciences, University of Auckland, Auckland 1023, New Zealand; 2 Department of Cell Biochemistry, Faculty of Biochemistry, Biophysics and Biotechnology, Jagiellonian University, Kraków 30-387, Poland

**Keywords:** Macrophage, Angiogenesis, Zebrafish, Tumour, Xenograft

## Abstract

Tumour angiogenesis has long been a focus of anti-cancer therapy; however, anti-angiogenic cancer treatment strategies have had limited clinical success. Tumour-associated myeloid cells are believed to play a role in the resistance of cancer towards anti-angiogenesis therapy, but the mechanisms by which they do this are unclear. An embryonic zebrafish xenograft model has been developed to investigate the mechanisms of tumour angiogenesis and as an assay to screen anti-angiogenic compounds. In this study, we used cell ablation techniques to remove either macrophages or neutrophils and assessed their contribution towards zebrafish xenograft angiogenesis by quantitating levels of graft vascularisation. The ablation of macrophages, but not neutrophils, caused a strong reduction in tumour xenograft vascularisation and time-lapse imaging demonstrated that tumour xenograft macrophages directly associated with the migrating tip of developing tumour blood vessels. Finally, we found that, although macrophages are required for vascularisation in xenografts that either secrete VEGFA or overexpress zebrafish *vegfaa*, they are not required for the vascularisation of grafts with low levels of VEGFA, suggesting that zebrafish macrophages can enhance Vegfa-driven tumour angiogenesis. The importance of macrophages to this angiogenic response suggests that this model could be used to further investigate the interplay between myeloid cells and tumour vascularisation.

## Abstract (precis)

**Summary:** Zebrafish embryonic macrophages associate with the distal tips of tumour xenograft blood vessels and are required for Vegfa-driven angiogenesis.

## INTRODUCTION

Angiogenesis has been a focus of cancer research due to its importance for tumour growth and metastasis (Hanahan and Weinberg, 2011; Presta et al., 1997; Huang et al., 2010). The tumour vasculature often appears as a disorganised network of permeable and irregularly shaped vessels and arises through a range of mechanisms, which include the induction of sprouting angiogenesis, endothelial progenitor-mediated vasculogenesis and the co-option of existing vessels (Krishna Priya et al., 2016). Many angiogenesis inhibitor drugs have targeted vascular endothelial growth factor (VEGF) signalling, a pathway that plays a key role in both developmental and disease-associated angiogenesis by stimulating the proliferation and migration of endothelial cells (Lee et al., 2015; Sennino and McDonald, 2012; Ye, 2016). However, VEGF inhibitors have had limited clinical success, with the cancer often relapsing after an initial period of remission (Carrato et al., 2013; Robert et al., 2011; Casanovas et al., 2005; Ferrara, 2010).

Inflammation can also drive tumour angiogenesis (Marelli et al., 2017), with the presence of leukocytes in the tumour region being positively correlated with levels of tumour vascularisation and associated with poor patient prognosis (Granot and Jablonska, 2015; Sionov et al., 2015; Mantovani et al., 2017). Macrophages and neutrophils can also promote angiogenesis in a variety of other settings, such as during developmental angiogenesis and in post-ischaemic neovascularisation (Fantin et al., 2010; Ohki et al., 2005). Macrophages have been shown to drive tumour vascularisation in mouse models (Piaggio et al., 2016; De Palma et al., 2003; Lin et al., 2006) and the depletion of tumour-associated macrophages results in an improved response to drugs targeting the VEGFR pathway, suggesting that macrophages aid in the escape from VEGFR inhibition (Shojaei et al., 2009; Zhang et al., 2010). Tumour-associated macrophages appear to stimulate angiogenesis by either direct or indirect mechanisms that increase the levels of pro-angiogenic factors (such as VEGF, HIF-1α and CCL18) (Guo et al., 2016; Lewis et al., 2016; Noy and Pollard, 2014; Deryugina and Quigley, 2010) or by transdifferentiation into endothelial-like cells that are capable of forming tubular structures (Asahara et al., 1997; Chen et al., 2009; Coukos et al., 2007). Neutrophils have also been shown to promote tumour angiogenesis, particularly via the production of MMP-9 (Nozawa et al., 2006; Uribe-Querol and Rosales, 2015; Ardi et al., 2009).

The embryonic zebrafish tumour xenograft model exploits the optical transparency of the zebrafish embryo and the availability of fluorescent blood vessel reporter lines to study the process of tumour angiogenesis. The model involves implanting tumour cells into the perivitelline space of a 2-day-old zebrafish embryo and observing the angiogenic response over the next 2 days (Nicoli and Presta, 2007). The blood vessels that grow into the xenograft have been shown to form an abnormal network of varying vessel morphology typical of mammalian tumours (Zhao et al., 2011a). The xenograft can be imaged *in vivo*, allowing observation of the mechanisms driving tumour vascularisation, such as sprouting angiogenesis, vascular co-option and endothelial cell migration (Zhao et al., 2011a,b), and can be used to screen for novel anti-angiogenesis drugs (Okuda et al., 2016). Despite the successes of this model, the precise role of the xenografted tumour cells and tumour-associated immune cells, with respect to the stimulation of angiogenesis, has not yet been determined. As zebrafish macrophages have previously been shown to be required for inflammatory lymphangiogenesis and to express pro-angiogenic *vegf* ligands (Okuda et al., 2015), this led us to investigate the role of macrophages in the zebrafish embryo tumour xenograft model of tumour angiogenesis.

In this study, we found that VEGFR-dependent angiogenesis occurs upon implantation of tumour cells or non-tumour cells into zebrafish embryos and that, although neutrophils and macrophages are recruited to these grafts, only macrophages have a role in tumour xenograft angiogenesis. Live-imaging analysis demonstrates that macrophages associate with developing tumour xenograft blood vessels, suggesting that they are directly mediating angiogenesis. We also showed that macrophages are required for angiogenesis when VEGFA/*vegfaa*-secreting cells are xenografted, but not in grafts with low levels of VEGFA secretion, suggesting that macrophages have a role in enhancing Vegfa-driven angiogenesis. These findings demonstrate that embryonic zebrafish xenografts can model macrophage-mediated angiogenesis and can be used to provide insights into the interface between innate immunity and tumour vascularisation.

## RESULTS

### Angiogenic and immune responses are observed upon graft implantation

**Fig. 1.**
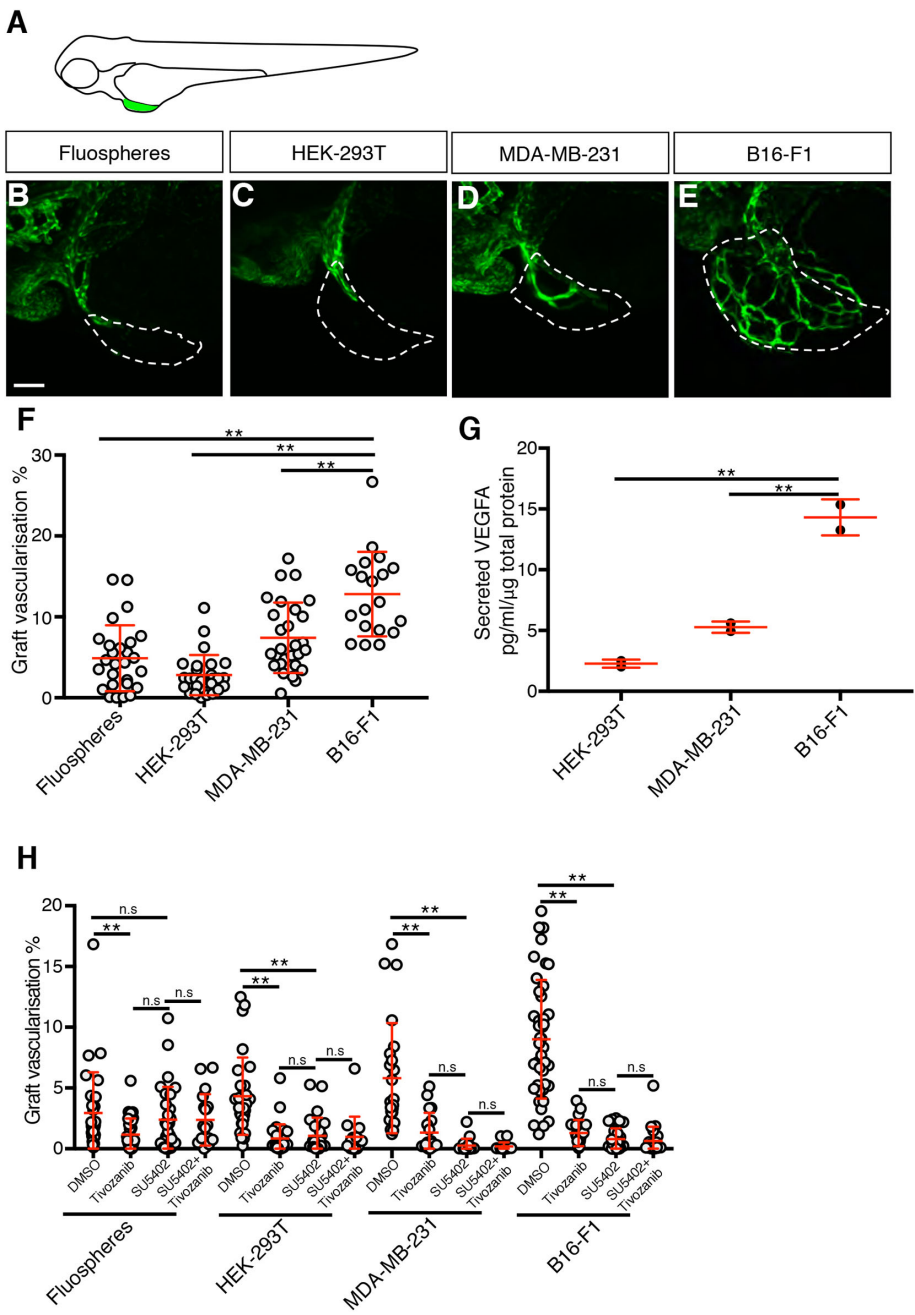
**Graft implantation induces an angiogenic response.** (A) Schematic indicating the location of the implanted graft (green) in the perivitelline space of a zebrafish embryo. (B-E) Confocal images of *kdrl:EGFP*-expressing blood vessels (green) in zebrafish embryo grafts (white dashed line) at 2 dpi. (F) Quantitation of graft vascularisation at 2 dpi, *n*>18. (G) Quantitation of secreted VEGFA levels, *n*=2. (H) Quantitation of graft vascularisation in embryos incubated in either 0.5% DMSO, 50 nM Tivozanib, 200 nM SU5402 or 50 nM Tivozanib+200 nM SU5402, *n*>14. Error bars represent s.d. n.s, *P*>0.05; **P<0.01 by one-way ANOVA. Scale bar: 50 µm.

To study the role of innate immune cells during tumour angiogenesis, we implanted either B16-F1 mouse melanoma cells, MDA-MB-231 human breast cancer cells, HEK-293T human embryonic kidney cells or abiotic Fluosphere beads into the perivitelline space of embryos at 2 days post-fertilisation (dpf) (Fig. 1A). We quantitated graft vascularisation by live imaging larvae at 2 days post-injection (dpi) and establishing the percentage volume of the graft that was occupied by GFP-expressing blood vessels. Using this method, we found the B16-F1 grafts displayed the highest level of vascularisation, followed by MDA-MB-231 cells. Surprisingly, both HEK-293T and Fluosphere grafts also induced vascularisation, albeit to a lower level than the cancer cell lines (Fig. 1B-F). The quantity of VEGFA secreted by the three cell lines was also assessed, and it was found that it positively correlated with their levels of vascularisation: B16-F1 cells secreted the highest levels of VEGFA, followed by the MDA-MB-231 cells and lastly the HEK-293T cells (Fig. 1G). In support of VEGFR signalling being required for graft angiogenesis, vascularisation in all three grafts was inhibited by the VEGFR inhibitor tivozanib (Fig. 1H) (Nakamura et al., 2006; Okuda et al., 2015). FGF signalling has also been implicated in zebrafish graft vascularisation (Nicoli et al., 2007), but treatment with the FGF/VEGFR co-inhibitor SU5402 (Sun et al., 1999), either alone or in combination with tivozanib, did not further inhibit graft vascularisation when compared to tivozanib treatment alone (Fig. 1H), suggesting that FGF signalling is not a major driver of angiogenesis in this model.

**Fig. 2.**
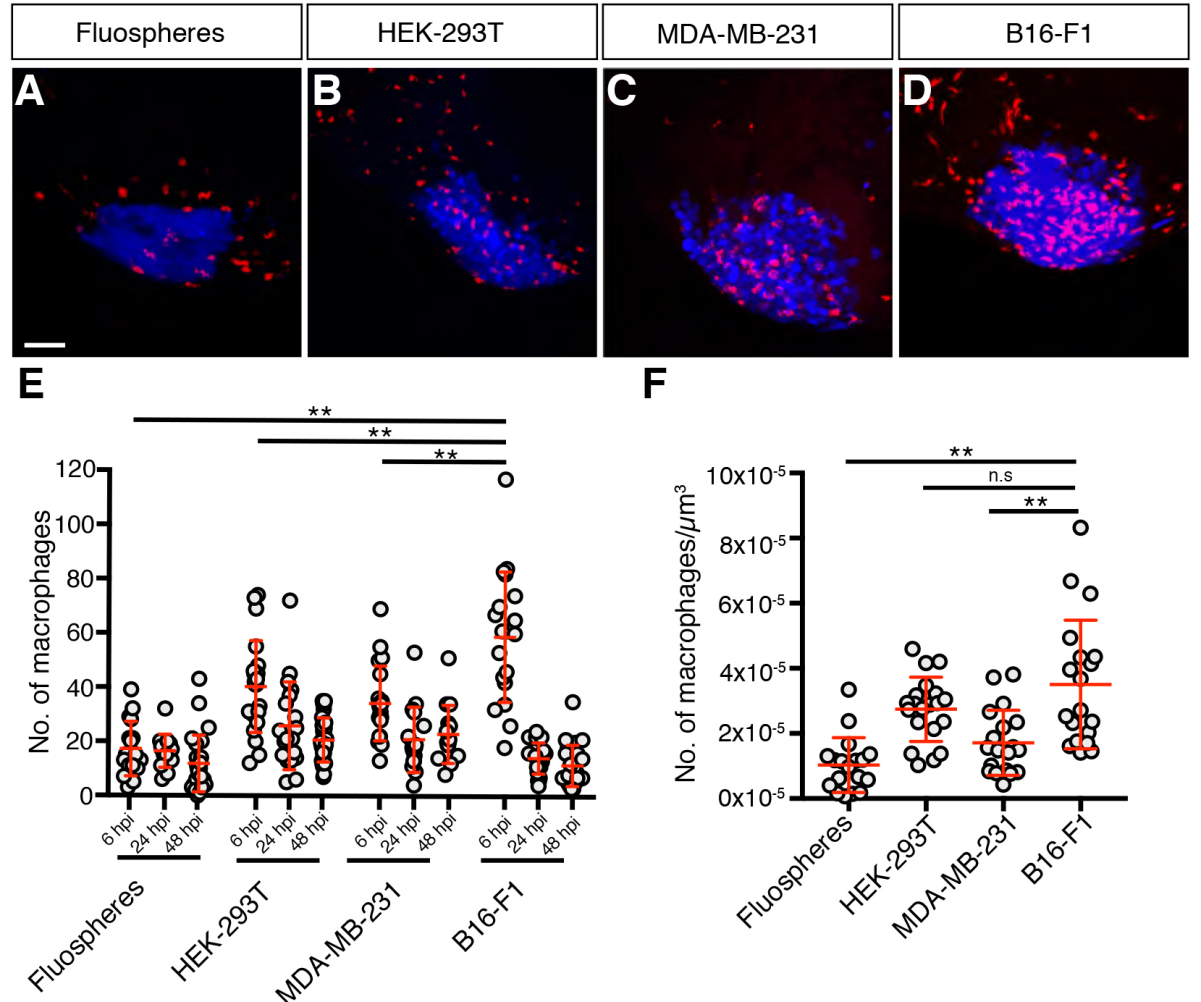
**Macrophages are recruited to grafts.** (A-D) Confocal images of *mpeg1:mCherry*-expressing macrophages (red) in zebrafish embryo grafts (blue) at 6 hpi. (E) Quantitation of graft-associated macrophages at 6, 24 and 48 hpi, *n*>14. (F) Quantitation of graft-associated macrophages normalised for graft volume at 6 hpi, *n*>16. Error bars represent s.d. n.s, *P*>0.05; **P<0.01 by one-way ANOVA. Scale bar: 50 µm.

We next assessed innate immune cell recruitment to the grafts by counting either *mpeg*-expressing macrophages (Ellett et al., 2011) or *mpx*-expressing neutrophils (Lieschke et al., 2001; Renshaw et al., 2006; Okuda et al., 2015) within the graft at 6 h post-injection (hpi), 24 hpi and 48 hpi. All graft types recruited both macrophages and neutrophils (Fig. 2 and Fig. S1), with macrophage numbers peaking at 6 hpi. B16-F1 grafts displayed the highest number of macrophages at this timepoint, with the MDA-MB-231 and HEK-293T xenografts displaying similar levels of macrophage recruitment, while the Fluosphere grafts had the lowest levels (Fig. 2). These findings were also observed when we normalised macrophage recruitment against graft volume, although the HEK-293T xenografts showed a level of macrophage recruitment more similar to the B16-F1 grafts by this measure (Fig. 2F).

### Macrophages contribute to tumour xenograft vascularisation

**Fig. 3.**
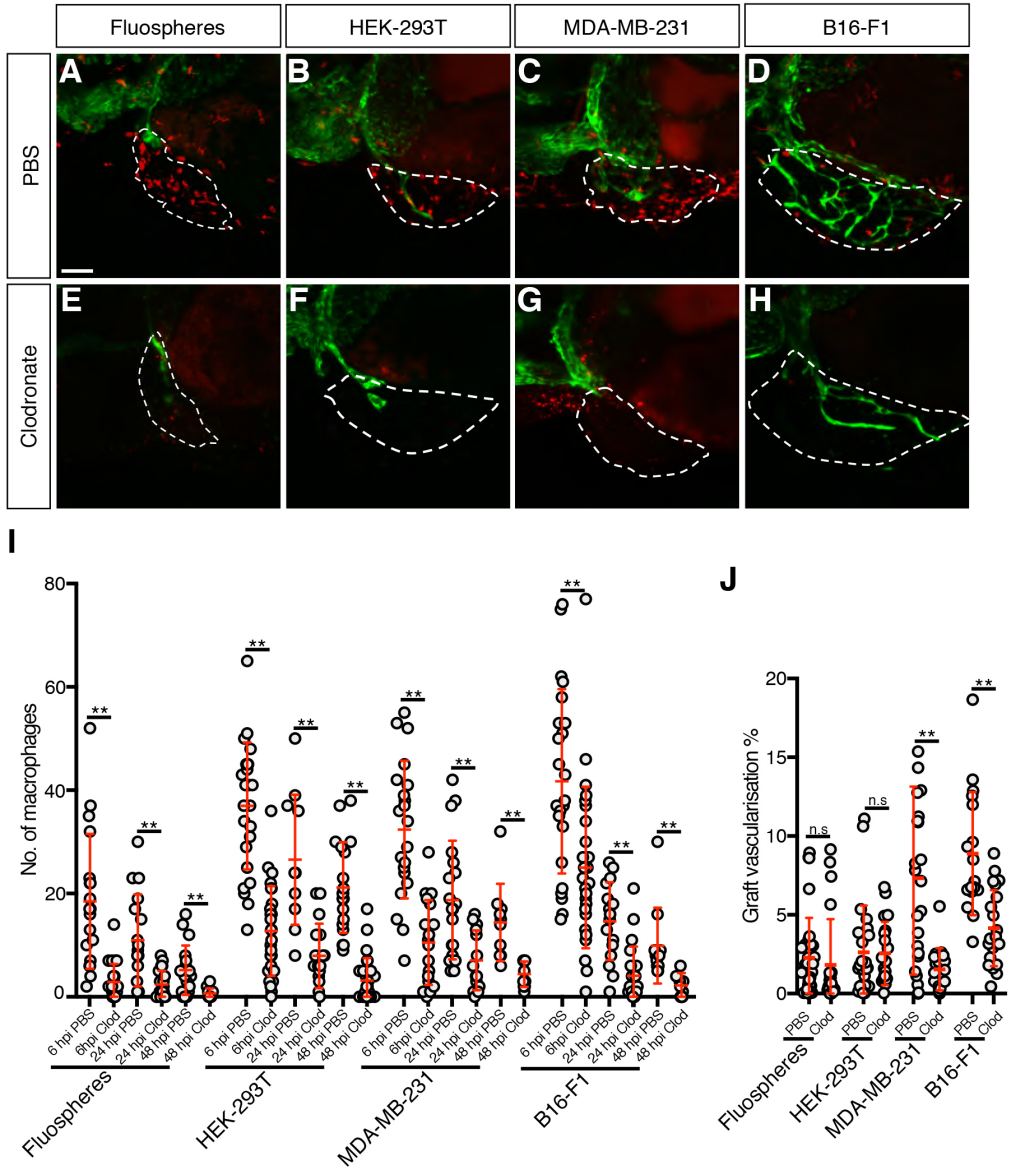
**Macrophages contribute to tumour xenograft vascularisation.** (A-H) Confocal images of *mpeg1:mCherry*-expressing macrophages (red) and *kdrl:EGFP*-expressing blood vessels (green) in zebrafish embryo grafts (white dashed line) at 2 dpi that have been injected with either PBS-containing liposomes (A-D) or clodronate-containing liposomes (E-H). (I) Quantitation of graft-associated macrophages at 6, 24 and 48 hpi with either PBS-containing or clodronate-containing liposomes, *n*>10. (J) Quantitation of graft vascularisation at 2 dpi, *n*>16. Error bars represent s.d. n.s, *P*>0.05; **P<0.01 by *t*-test. Scale bar: 50 µm.

Given that all graft types could induce both immune cell recruitment and vascularisation, we wondered whether leukocytes had a role in the angiogenic response. We conducted clodronate-mediated macrophage ablation to specifically induce macrophage death in grafted larvae (Hall et al., 2018; Astin et al., 2017; Carrillo et al., 2016; Lai et al., 2017). The efficacy of macrophage reduction was assessed by measuring the difference in the number of graft-associated macrophages in larvae injected with clodronate-containing liposomes and larvae injected with PBS-containing liposomes. Clodronate-mediated macrophage ablation produced a reduction in graft-associated macrophages of at least 40% by 6 hpi, 60% by 24 hpi and 70% by 48 hpi (Fig. 3A-I), did not alter the level of neutrophil recruitment to the graft (Fig. S2A), and resulted in a 50% reduction in tumour vascularisation in both the MDA-MB-231 and B16-F1 xenografts at 48 hpi (Fig. 3A-H,J). In contrast, both HEK-293T and Fluosphere grafts displayed no difference in vascularisation at 48 hpi when subjected to macrophage ablation (Fig. 3A-H,J), suggesting that macrophages only have a role in the vascularisation of tumour xenografts.

In the embryos subjected to macrophage ablation, the main difference in the tumour xenograft vasculature was a reduction in the number of vessels growing inside the tumour region and the depth to which these vessels penetrated the tumour mass compared to the control fish. This was most obvious in the B16-F1 xenografts, where the control tumours often had a rich network of blood vessels growing through the entire xenograft, while the embryos subjected to ablation only showed a few vessels forming in a localised part of the xenograft (Movies 1 and 2).

To confirm the effect of macrophage ablation on tumour xenograft vascularisation, we also employed nitroreductase-mediated macrophage ablation on the MDA-MB-231-xenografted *Tg(mpeg1:NTR:mcherry)* larvae by treating them with 5 mM metronidazole (Okuda et al., 2015; Petrie et al., 2014). Graft-associated macrophages were reduced by 40% at 6 hpi and 60% at 24 hpi, and xenograft vascularisation was reduced by more than 40% in the embryos incubated in metronidazole compared with the embryos incubated in DMSO (Fig. S3). Overall, using both clodronate- and nitroreductase-mediated ablation, we have shown that macrophages drive vascularisation of tumour xenografts in zebrafish embryos.

As neutrophil recruitment was also observed in the tumour xenografts, we sought to assess the contribution of neutrophils by observing the effects of their removal via nitroreductase-mediated ablation in *Tg(mpx:NTR:mCherry)* larvae. Despite reducing graft-associated neutrophil numbers by 35% at 6 hpi and 75% at 1 dpi, no significant difference was seen in graft vascularisation at 2 dpi (Fig. S4), suggesting that neutrophils do not have a significant role in graft vascularisation. Importantly, metronidazole, the pro-drug used in nitroreductase-mediated ablation, did not have any effect on the level of macrophage or neutrophil recruitment or on graft vascularisation when administered to larvae lacking the nitroreductase enzyme (Fig. S2B-D).

### Macrophages associate with growing tumour vessels

**Fig. 4.**
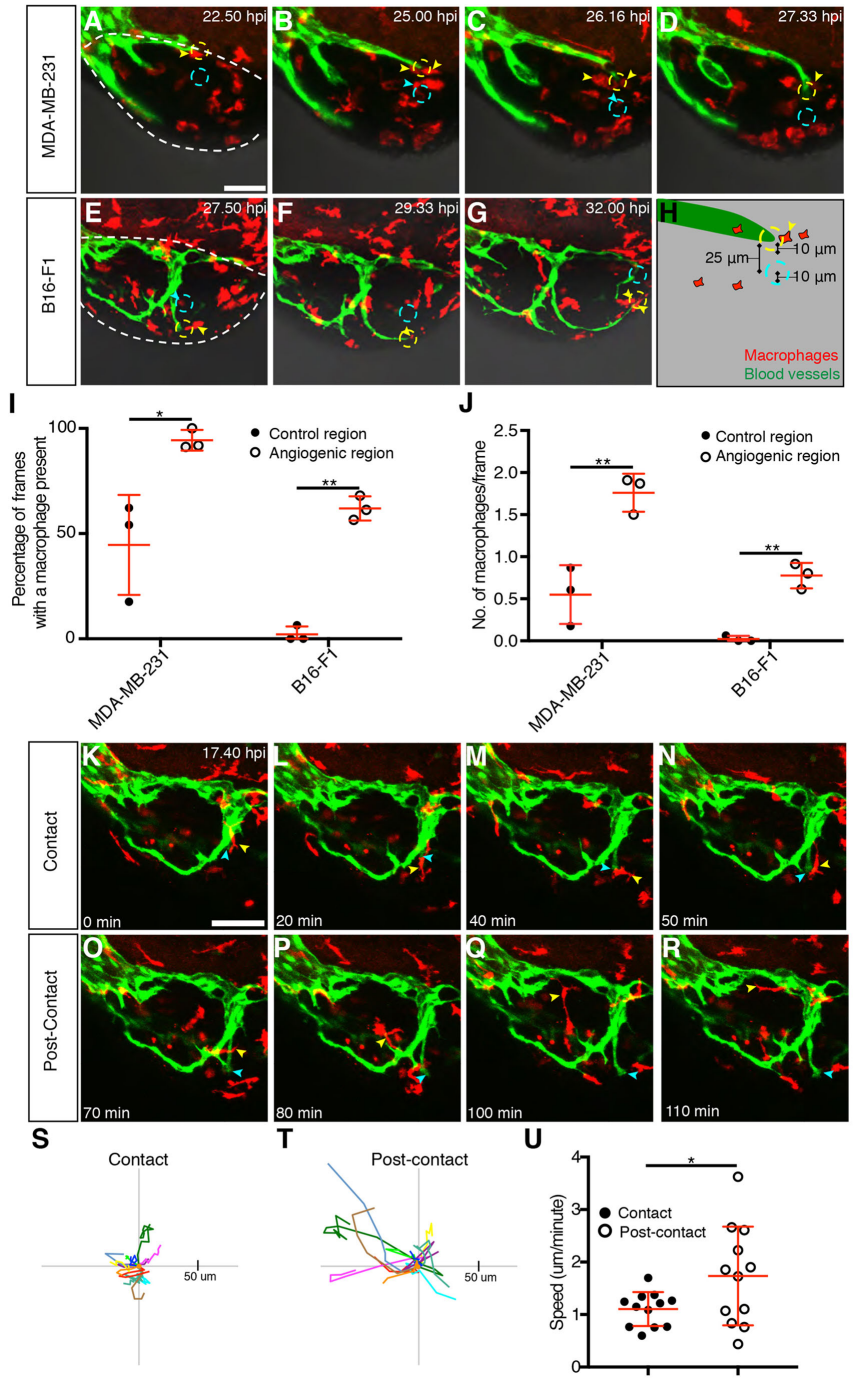
**Macrophages associate with developing xenograft vessels.** (A-G) Still images showing an MDA-MB-231 xenograft (A-D, Movie 3) or a B16-F1 xenograft (E-G, Movie 4) in an embryo with *mpeg1:mCherry*-expressing macrophages (red) and *kdrl:EGFP*-expressing blood vessels (green). Individual macrophages associated with the angiogenic region (yellow dashed circle) are indicated with yellow arrowheads, while macrophages associated with the control region (cyan dashed circle) are indicated with cyan arrowheads. The tumour region is outlined by a white dashed line in A and E. (H) Schematic demonstrating the positioning of the 10-μm angiogenic region (dashed yellow outline) at the tip of the blood vessel (green) and the control 10-μm control region (dashed cyan outline). (I) Quantitation of the percentage of frames during which a macrophage was observed at either an angiogenic region or a control region in MDA-MB-231 and B16-F1 xenografts, *n*=3. (J) Mean number of macrophages observed during each frame, in either the angiogenic region or the control region, in MDA-MB-231 and B16 F1 xenografts, *n*=3. (K-R) Still images showing a B16-F1 xenograft (Movie 4) in an embryo with *mpeg1:mCherry*-expressing macrophages (red) and *kdrl:EGFP*-expressing blood vessels (green). An individual macrophage (yellow arrowhead) is tracked for 1 h 10 min during (K-N) and after (O-R) associating with the distal tip of a growing vessel (indicated by a cyan arrowhead). (S,T) Macrophage migration tracks of 12 macrophages during their period of contact with the tip of a growing vessel (S) and of the same macrophages once they leave the vessel tip (T). Each macrophage is depicted with the same colour in both S and T and they were tracked for identical periods of time during contact and post-contact. (U) Quantitation of macrophage migration speed during and after the period of contact with the tip of a growing vessel, *n*=12. Error bars represent s.d. *P<0.05, **P<0.01 by *t*-test. Scale bars: 50 µm.

Given that macrophages have a role in angiogenesis within tumour xenografts, we sought to observe their behaviour during the angiogenic process. Time-lapse imaging of angiogenesis in the MDA-MB-231 and B16-F1 tumour xenografts was conducted from 8 hpi and we consistently observed macrophages interacting with the blood vessels growing within the xenograft (Fig. 4A-G, Movies 3 and 4). Specifically, we saw an increased presence of tumour-xenograft-associated macrophages at the distal tips of xenograft blood vessels.

To confirm the significance of the macrophage–vessel interactions, we quantitated the association of graft macrophages with the vascular tips. We measured macrophage presence in a 10 μm circle centred at the tip of a growing vessel (angiogenic region) and compared this with a control region of the same size located at a constant distance from the vascular tip (Fig. 4A-H, described in detail in the Materials and Methods). We found that macrophages were more commonly present at or near the tips of these developing tumour vessels than in the avascular control regions (Fig. 4I) and that there were also more macrophages located at the tips of the vessels compared to avascular regions (Fig. 4J), suggesting that macrophages play a direct role in xenograft angiogenesis. We defined a ‘tip cell’ macrophage as one that associated with a distal tip of a developing graft blood vessel for at least 40 min, and identified 12 tip cell macrophages from our time-lapse movies of MDA-MB-231 and B16-F1 grafts. Tip cell macrophages associated with the graft blood vessel for an average of 69 min and, during this time, ceased active migration and maintained contact with the vessel tip, only resuming normal macrophage migratory behaviour once they had dissociated from the vessel (Fig. 4K-U). We also tracked tip cell macrophages both before and after their association with a vessel and this revealed that only 3/11 tip cell macrophages made an association with another blood vessel tip, suggesting that there is a large pool of potential ‘tip cell macrophages’ within the graft.

### Macrophages are required for effective angiogenesis induced by graft-expressed *vegfaa*

**Fig. 5.**
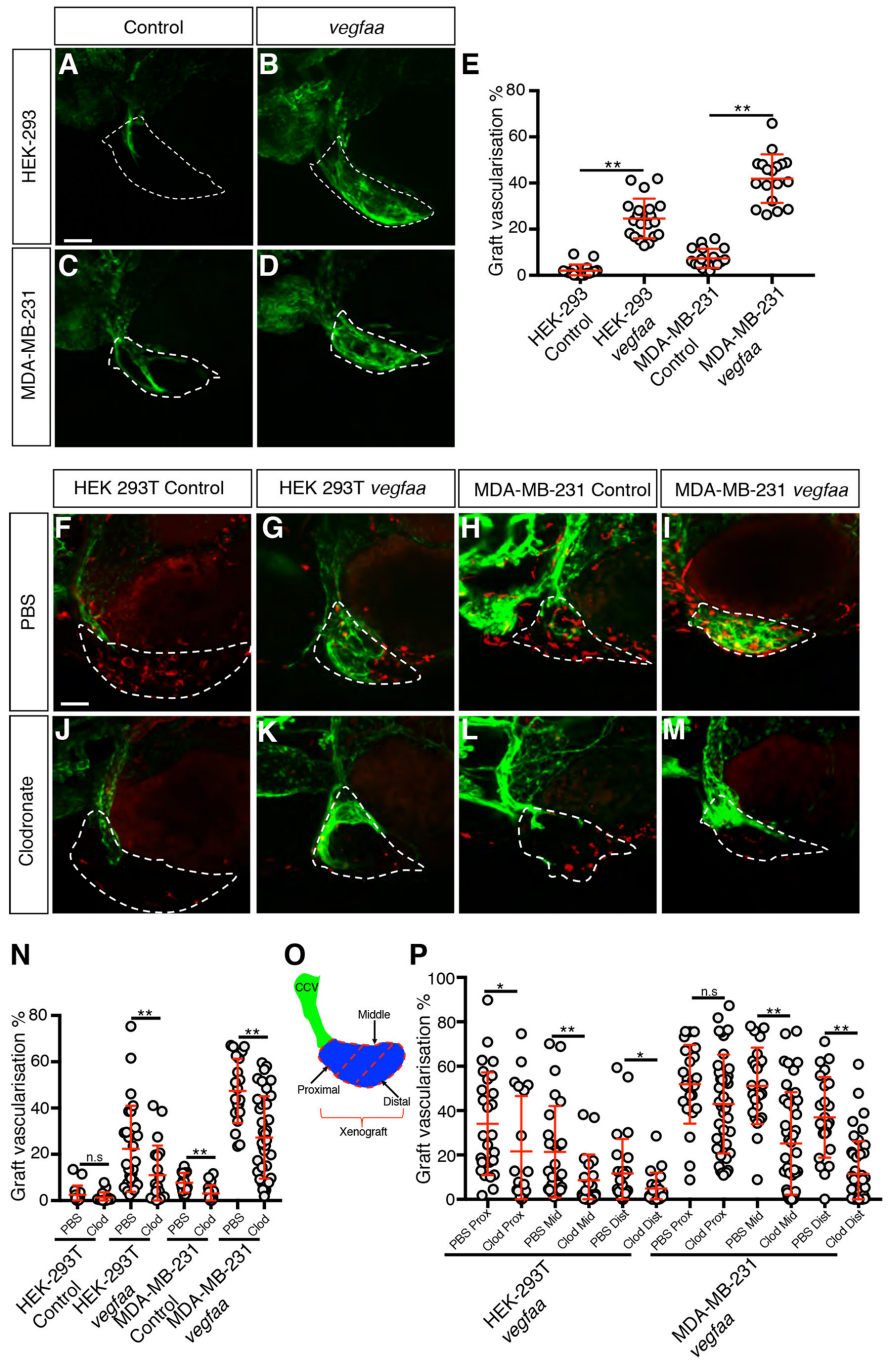
**Macrophages are required for effective *vegfaa*-driven angiogenesis.** (A-D) Confocal images taken at 2 dpi of *kdrl:EGFP*-expressing vessels (green) in zebrafish embryos implanted with either HEK-293T (A,B) or MDA-MB-231 (C,D) xenografts (white dashed line) transfected with either a control expression vector (A,C) or a *vegfaa*-expression vector (B,D). (E) Quantitation of graft vascularisation at 2 dpi, *n*>17. (F-M) Confocal images taken at 2 dpi of *mpeg1:mCherry*-expressing macrophages (red) and *kdrl:EGFP*-expressing blood vessels (green) in zebrafish embryos implanted with either HEK-293T or MDA-MB-231 xenografts (white dashed lines) transfected with either a control expression vector (F,H,J,L) or a *vegfaa*-expression vector (G,I,K,M) that have been injected with either PBS-containing liposomes (F-I) or clodronate-containing liposomes (J-M). (N) Quantitation of graft vascularisation at 2 dpi in embryos injected with either PBS-containing or clodronate-containing liposomes, *n*>16. (O) Schematic demonstrating how the proximal, middle and distal sections (marked by dashed red lines) of the xenograft (blue) were determined by their location with respect to the CCV (green). (P) Quantitation of vascularisation at 2 dpi in the proximal, middle and distal regions of *vegfaa*-expressing HEK-293T or MDA-MB-231 xenografts implanted into embryos injected with either PBS-containing or clodronate-containing liposomes, *n*>19. Error bars represent s.d. n.s, *P*>0.05; *P<0.05, **P<0.01 by *t*-test. Scale bars: 50 µm.

As the cancer cells in our xenografts express VEGFA (Fig. 1G) and xenograft vascularisation is sensitive to inhibition of VEGFR signalling (Fig. 1H), this led us to hypothesise that the xenograft-associated macrophages can potentiate tumour angiogenesis that is driven by xenograft-supplied VEGFA. To test this hypothesis, we developed both HEK-293T and MDA-MB-231 cell lines that express a zebrafish ortholog of *VEGFA* (*vegfaa*). The cell lines transfected with the *zf*-*vegfaa*-expression vector were confirmed to express *vegfaa* by reverse-transcription PCR (RT-PCR) (Fig. S5A) and, when xenografted in zebrafish embryos, both HEK-293T–*vegfaa* and MDA-MB-231–*vegfaa* xenografts displayed a level of vascularisation significantly higher than the control lines (Fig. 5A-E), while macrophage recruitment in the *vegfaa-*expressing xenografts was similar to the control (Fig. S5B-G). Clodronate-mediated macrophage ablation was shown to be effective in xenografts of both *vegfaa*-expressing cell lines, resulting in a reduction of graft-associated macrophages of at least 50% at 6 hpi and 80% at 24 hpi (Fig. S5H,I). Importantly, macrophage ablation caused a 40% reduction in the level of vascularisation in both HEK-293T and MDA-MB-231 *vegfaa*-expressing xenografts (Fig. 5F-N), supporting our hypothesis that macrophages are required for effective angiogenesis in grafts expressing *vegfaa*.

The graft vessels predominantly form by sprouting from the common cardinal vein (CCV). We found that the *vegfaa*-expressing xenografts appeared to induce a very strong angiogenic response, with vessels present throughout the volume of the xenograft. By contrast, in the embryos subjected to macrophage ablation, xenograft vessels were proximal to the CCV and either did not extend distally into the tumour or only grew into the superficial layers of the xenograft (Movies 5-8). The extent of graft vascularisation was quantitated by dividing the graft into equal thirds (proximal to the CCV, middle and distal to the CCV) and quantitating the level of vascularisation within each of these subsections (Fig. 5O). We found that, while the grafts in macrophage-ablated embryos had similar levels of proximal vascularisation, the middle and distal thirds of the graft had significantly lower levels of vascularisation when compared to control (Fig. 5P).

### Macrophages are not required for vascularisation of MDA-MB-231 xenografts depleted of VEGFA

**Fig. 6.**
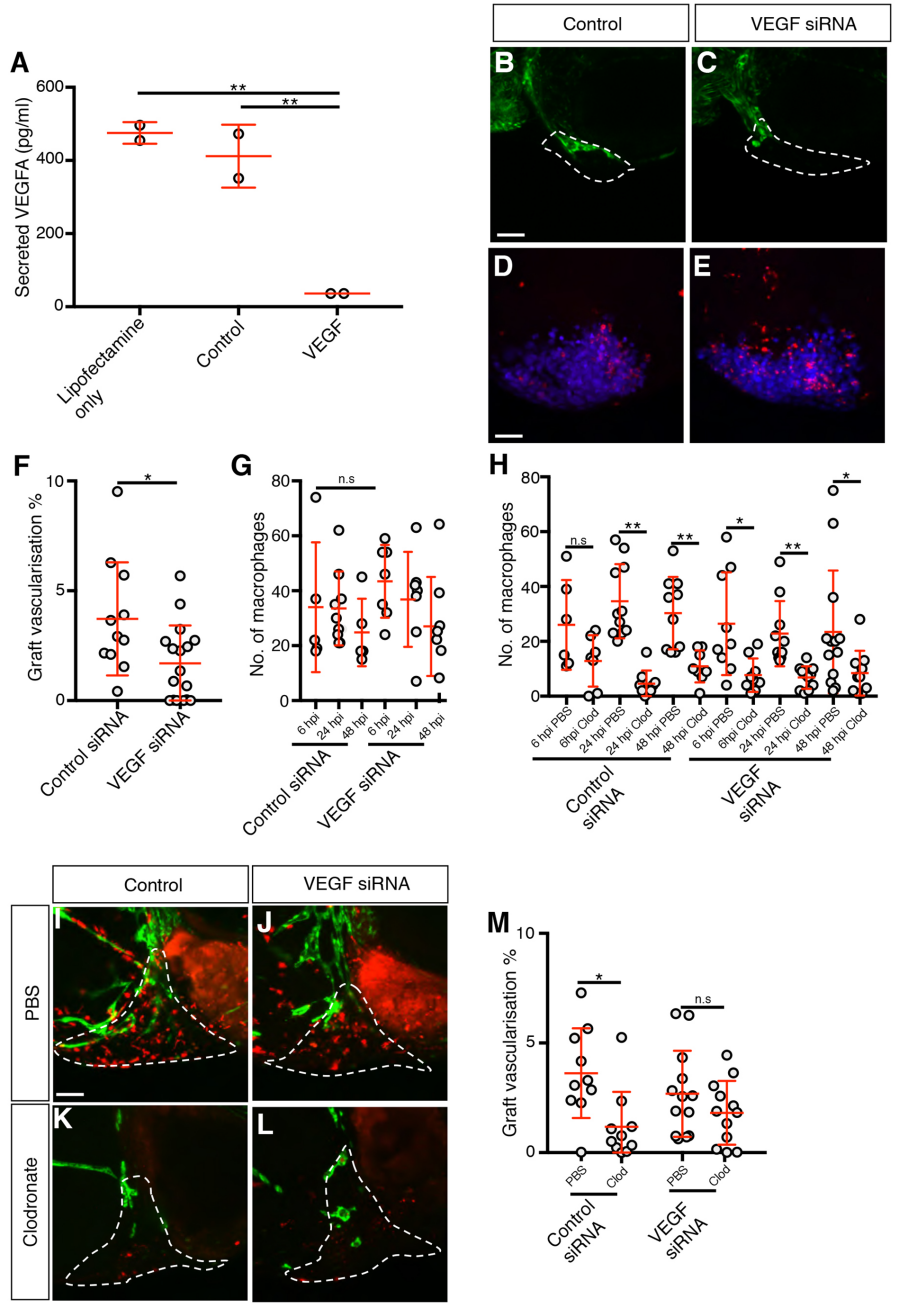
**Macrophages are not required for vascularisation in MDA-MB-231 xenografts depleted of VEGFA.** (A) Quantitation of secreted VEGFA levels in 2×10^5^ siRNA-treated cells, *n*=2. (B,C) Confocal images taken at 2 dpi of *kdrl:EGFP*-expressing vessels (green) in zebrafish embryos implanted with MDA-MB-231 xenografts (white dashed line) transfected with either control (B) or *VEGFA* siRNA (C). (D,E) Confocal images taken at 6 hpi of *mpeg1:mCherry*-expressing macrophages (red) and MDA-MB-231 xenografts (blue). (F,G) Quantitation of graft vascularisation at 2 dpi, *n*>10 (F), and of graft-associated macrophages at 6, 24 and 48 hpi, *n*>4 (G). (H) Quantitation of graft-associated macrophages at 6, 24 and 48 hpi, in embryos injected with either PBS-containing or clodronate-containing liposomes, *n*>5. (I-L) Confocal images taken at 2 dpi of *mpeg1:mCherry*-expressing macrophages (red) and *kdrl:EGFP*-expressing blood vessels (green) in zebrafish embryos implanted with MDA-MB-231 xenografts (white dashed lines) transfected with either control (I,K) or *VEGFA* (J,L) siRNA that have been injected with either PBS-containing liposomes (I,J) or clodronate-containing liposomes (K,L). (M) Quantitation of graft vascularisation at 2 dpi in embryos injected with either PBS-containing or clodronate-containing liposomes, *n*>9. Error bars represent s.d. n.s, *P*>0.05; *P<0.05; **P<0.01 by either one-way ANOVA (A) or *t*-test (F-H,M). Scale bars: 50 µm.

To determine whether the role of macrophages in graft vascularisation was confined to angiogenesis driven by xenograft-supplied VEGFA, we used siRNA to knock down *VEGFA* in MDA-MB-231 cells. We achieved an 85% reduction in the levels of secreted VEGFA in siRNA-treated cells (Fig. 6A) which resulted in a 50% reduction in graft vascularisation when compared to control siRNA-treated cells (Fig. 6B,C,F), while the levels of graft-associated macrophages remained unchanged (Fig. 6D,E,G). When macrophages were ablated with clodronate, the level of graft vascularisation remained unchanged (Fig. 6H-M), suggesting that macrophages are not required for angiogenesis in tumour xenografts with low levels of VEGFA.

## DISCUSSION

The zebrafish embryo tumour xenograft model has been developed as an alternative to rodent and chick xenografts for the investigation of tumour angiogenesis (Brown et al., 2017). The model was first described in 2007 (Nicoli et al., 2007) and, since then, it has been used to further the knowledge of the mechanisms of tumour angiogenesis: identifying genes of interest, determining the cellular processes involved in tumour angiogenesis and elucidating the functions carried out by tumour blood vessels (Vlecken and Bagowski, 2009; Zhao et al., 2016, 2011b). The main strengths of this model are the ability to image tumour angiogenesis *in vivo* and the applicability for drug screening (Harfouche et al., 2009; Yang et al., 2014a,b; Muthukumarasamy et al., 2016; Zhao et al., 2011b, 2016). While this model has gained popularity, the exact mechanisms that underpin xenograft angiogenesis in zebrafish are still unclear. In this study, we found that the angiogenic response observed in zebrafish tumour xenografts has an inflammatory-driven component and that macrophages are required for effective angiogenesis within VEGFA/*vegfaa*-producing xenografts.

We used 3D-image analysis of the graft to quantitate tumour vascularisation (Astin et al., 2014), which allowed us to determine the angiogenic potential of different grafts and the effects of different treatment strategies on the angiogenic response. In the original description of this model, Nicoli et al., (2007) found that certain non-tumour mammalian cell lines do not induce an angiogenic response; however, we observed a modest angiogenic response in non-tumour cell lines and even upon implantation of an abiotic Fluosphere graft. This discrepancy can be explained by the fact that we performed a quantitative analysis of graft vascularisation, whereas previous studies had used less-sensitive qualitative analyses to measure the angiogenic effects of implantation of non-tumour cell lines (Nicoli et al., 2007). Because of this, we propose that quantitative analyses, such as the one described in this study, should be considered in future studies using this model.

Our observations of macrophage and neutrophil recruitment are consistent with other studies using this model (Yang et al., 2013; He et al., 2012; Sanderson et al., 2015). We observed macrophage and neutrophil recruitment within the tumour and non-tumour xenografts, as well as in the Fluosphere grafts. This recruitment appeared to follow a pattern of increasing leukocyte numbers over the first 6 hpi (data not shown) and a subsequent decrease in macrophage numbers over the next 2 dpi, demonstrating early and high levels of leukocyte recruitment to the grafts. We used both clodronate liposomes and nitroreductase-mediated cell ablation to reduce macrophage number, and obtained quantitative measurements in xenograft vascularisation. We found that macrophages were required for effective vascularisation of xenografts that expressed either VEGFA or zebrafish *vegfaa*, with a dramatic decrease in vascularisation observed when macrophages were ablated from either VEGFA-expressing xenografts (B16-F1 or MDA-MB-231) or in xenografts that overexpressed *vegfaa*. Our data support previous evidence of macrophage involvement in zebrafish graft vascularisation that was based on qualitative observations of reduced vascularisation upon inhibition of the myeloid lineage using a *spi1b* morpholino (He et al., 2012). Our findings also support studies in murine models, where the addition or deletion of macrophages results in a respective increase or decrease in tumour vascularisation (De Palma et al., 2003, 2005; Lin et al., 2006), and human tumours, where macrophage presence has been correlated with increased tumour angiogenesis and poor patient prognosis (Heusinkveld and van der Burg, 2011).

The high level of macrophages within the poorly vascularised HEK-293T xenografts indicates that the presence of graft-associated macrophages is not sufficient to induce high levels of angiogenesis. We also demonstrate that macrophages are only required for graft vascularisation when the graft cells express VEGFA/*vegfaa*: in contrast to the VEGFA-expressing B16-F1 and MDA-MB-231 tumour xenografts, in either Fluosphere or HEK-293T grafts, which respectively express either no VEGFA or low levels of VEGFA, macrophages had no detectable role in graft vascularisation. In addition, we show that macrophages are not required for vascularisation of MDA-MB-231 xenografts that are depleted in VEGFA levels, while, conversely, macrophages are required for vascularisation of HEK-293T grafts that overexpress *vegfaa*. Taken together, these data suggest that there may be differences in the angiogenic potential of graft macrophages between the different graft types and that this relates to the differing levels of VEGFA secretion from the grafts. One possibility is that there is a threshold level of VEGFA/*vegfaa* that is required for macrophages to become pro-angiogenic and, in support of this, there is evidence that VEGFA can influence the activation state of mammalian macrophages (Kloepper et al., 2016; Wheeler et al., 2018). Another, perhaps more likely, possibility is that a base level of graft angiogenesis is required before any pro-angiogenic macrophage function can become evident in our assay; pro-angiogenic macrophages can enhance vessel migration and promote vascularisation but they may be unable to stimulate new vessel sprouts *de novo* and therefore a macrophage-extrinsic angiogenic stimulus is required (such as graft-supplied VEGFA/*vegfaa*) before macrophages can promote vessel growth.

It is known that macrophages can exist in a diverse range of phenotypes, with the broadest classification dividing them into M1-like (which are more oriented towards a pathogen-fighting role) and M2-like (more oriented towards a tissue repair and immunosuppressive role) categories (Albini et al., 2018). The pro-angiogenic macrophages that are present in tumours are generally of the M2-like category, although they display a diversity not entirely reflective of the M1/M2 dichotomy and exist in subcategories such as the Tie2-expressing macrophages, which have been shown to be highly adept at promoting angiogenesis and other pro-tumour functions (Squadrito and De Palma, 2011). It is possible that cytokines produced by tumour xenografts (such as M-CSF, IL-10 and TGF-β) activate macrophages that migrate to the xenograft, and contribute to their polarisation to a pro-angiogenic phenotype (Galdiero et al., 2013). In zebrafish, embryonic macrophages have recently been demonstrated to play a role in both developmental and wound angiogenesis, although, surprisingly, these macrophages appear to be identified by the classical M1 marker Tnfα, highlighting the complexity of pro-angiogenic macrophage phenotypes (Gerri et al., 2017; Gurevich et al., 2018). The identity of the pro-angiogenic macrophages in this model could be investigated by the use of zebrafish transgenics that differentially label macrophages of different phenotypes (Kanther et al., 2011; Nguyen-Chi et al., 2015; Gerri et al., 2017).

The mechanism by which the pro-angiogenic zebrafish macrophages enhance xenograft vascularisation is unclear. Our time-lapse imaging data suggest that the role of macrophages involves their direct interaction with the developing blood vessel; we observed that macrophages frequently associated with the distal tips of developing xenograft blood vessels. In addition, our observation of limited endothelial cell penetration into the tumour region upon the ablation of macrophages suggests that macrophages may be assisting with the development of blood vessels through the tumour and has parallels to a recent report demonstrating that zebrafish larval macrophages associate with blood vessel tips at wounds and are required for neo-angiogenesis during wound healing (Gurevich et al., 2018). Potential mechanisms by which tumour macrophages can stimulate blood vessel growth include the secretion of pro-angiogenic factors (such as Vegfa, Egf, Fgf2, Cxcl8 and Cxcl12) to promote the proliferation and migration of endothelial cells through the graft and also the secretion of enzymes that stimulate the breakdown of the extracellular matrix (ECM) (such as uPA or Mmp2 and 9) (Albini et al., 2018). Our data support both of these possible mechanisms, whereby zebrafish pro-angiogenic macrophages secrete angiogenic factors (such as Vegfa) and/or allow ECM breakdown to drive angiogenesis in grafts. To further elucidate which of these mechanisms are employed by macrophages in this model, a macrophage-specific knockout of these pro-angiogenic pathways could be employed (Ablain et al., 2015).

Finally, we show that macrophages are not required for all VEGFR-mediated graft angiogenesis as the limited vascularisation of either HEK-293T grafts, which express low levels of VEGFA, or Fluospheres, which express no VEGFA, is sensitive to VEGFR inhibition but is not sensitive to macrophage ablation. We propose that the VEGFR-dependent vascularisation of Fluosphere grafts is likely due to the local release of VEGFR ligands from tissue damaged during graft implantation; various cells, including keratinocytes, are known to secrete VEGF ligands upon wounding (Brown et al., 1992; Kishimoto et al., 2000; Failla et al., 2000) and an upregulation of *vegfaa* has been observed in zebrafish wounds and granulomas (Gurevich et al., 2018; Marín-Juez et al., 2016; Oehlers et al., 2015). However, as discussed previously, the angiogenic response to Fluosphere and HEK-293T grafts does not require macrophage involvement, either due to a lack of pro-angiogenic macrophages within these grafts and/or because a higher level of extrinsic VEGF signalling is required before macrophage angiogenic function becomes evident.

Our study demonstrates that the angiogenic response observed within zebrafish VEGFA/Vegfaa*-*secreting xenografts requires a macrophage-driven inflammatory response. This expands the utility of this model as it can now be used to investigate the mechanisms by which macrophages can promote tumour angiogenesis. A greater understanding of these processes could help in the development of treatments for pathologies characterised by inflammatory angiogenesis, such as tumour angiogenesis, choroidal neovascularisation, arthritis and inflammatory bowel disease (Pollard, 2009). Finally, this study also has implications on the use of this model as a tumour angiogenesis assay: any discoveries made regarding angiogenesis using this model must be interpreted in the context of the inflammatory nature of the angiogenic response.

## MATERIALS AND METHODS

### Zebrafish husbandry and maintenance

All zebrafish (*Danio rerio*) strains were maintained under standard husbandry conditions and all studies carried out were approved by the University of Auckland Animal Ethics Committee. The following transgenic lines were used in this study: *Tg(mpeg1:Gal4FF)^gl25^* (Ellett et al., 2011), *Tg(UAS-E1b:nsfB-mCherry)^c264^* (Davison et al., 2007), *Tg*(-8. *mpx:*KalTA4)*^gl28^* (Okuda et al., 2015), *Tg(kdrl:EGFP)^s843^* (Jinn et al., 2005), *Tg(lyz:EGFP)^nz117^* (Hall et al., 2007) and *Tg(mpeg1:EGFP)^gl22^* (Hall et al., 2013). For convenience, the *Tg(mpeg1:Gal4FF)^gl25^*;*Tg(UAS-E1b:nsfB-mCherry)^c264^* line is referred to as *mpeg1:NTR:mCherry* and the *Tg*(-8.*mpx:*KalTA4)*^gl28^*;*Tg(UAS-E1b:nsfB-mCherry)^c264^* line is referred to as *mpx:NTR:mCherry*.

### Tissue culture

All cell lines were maintained at 37°C and 5% CO_2_, and passaged at 95-100% confluence. The following cell lines were used in this study: MDA-MB-231-luc D3 H2LN, B16-F1 and HEK-293T. B16-F1 and HEK-293T were originally obtained from the ATCC, while MDA-MB-231 cells were obtained from Caliper Life Sciences. MDA-MB-231 and B16-F1 cells were grown in MEM-alpha media, 10% FBS (Gibco) and 1% pen/strep. HEK-293T cells were grown in low glucose, pyruvate DMEM, 10% FBS (Gibco) and 1% pen/strep. All cell lines were tested to be Mycoplasma-negative using the MycoAlert™ Mycoplasma detection kit (Lonza).

### VEGFA secretion assay

Total protein content of the cells was determined by conducting a Pierce™ BCA Protein Assay using the Thermo Scientific kit and protocol. VEGFA secretion was detected by removing a 25 μl sample of media from a confluent 4 cm^2^ dish (containing 500 µl of media) and running it on either the Mouse or Human Cytokine Magnetic bead panels (MILLIPLEX^®^). The concentration of VEGFA in the well media and total protein content for each cell line was calculated for each well analysed, and VEGFA secretion was divided by protein content to produce a value of VEGFA secreted concentration with respect to total protein expression for each cell line, and the mean between the two samples was plotted on the graph for each cell line.

### *vegfaa* transfection

Full-length zebrafish *vegfaa* cDNA was isolated by TRIzol-extraction (Ambion) of RNA from a 1 dpf zebrafish followed by RT-PCR with Platinum™ Pfx DNA polymerase (Invitrogen) using zebrafish *vegfaa* primers (Forward: 5′-GCTAGCATGAACTTGGTTGTTTATTT-3′, Reverse: 5′-GCGGCCGCTCATCTTGGCTT-3′). *vegfaa* was cloned into the mammalian expression vector pIRES-P (Hobbs et al., 1998), which contains the puromycin-resistance gene. HEK-293 or MDA-MB-231 cells were transfected using Lipofectamine 2000 (Invitrogen) and transfected cells were subsequently maintained in media containing 2 µg/ml puromycin.

### *VEGFA* siRNA knockdown

siRNA knockdown was performed on MDA-MB-231 cells using an anti-VEGF siRNA (Thermo Fisher Scientific, cat.: 4392420), with scrambled siRNA (Thermo Fisher Scientific, cat.: 4390843) as the negative control. A total of 150 pmol of siRNA and 7.5 µl of Lipofectamine RNAiMAX (Invitrogen) were added to 500 µl Opti-MEM in one well of a 6-well plate and incubated at room temperature for 20 min. 200,000 MDA-MB-231 cells in 2.5 ml of supplemented media (without antibiotics) were added to the well and incubated at 37°C. After 24 h the cells were either harvested for implantation or the transfection media was replaced with serum-free media in order to assess VEGF knockdown at 48 h.

### RT-PCR

RNA was extracted from cell lines using TRIzol (Ambion). Superscript III Reverse Transcriptase (Invitrogen) was used to synthesise cDNA, and RT-PCR was conducted using the zebrafish *vegfaa* primers described above and human *GAPDH* primers as a control (Forward: 5′-ACGGGAAGCTTGTCATCA-3′, Reverse: 5′-TGGACTCCACGACGTACTCA-3′).

### Xenotransplantation

Cells were trypsinised, centrifuged and labelled by resuspending in a 2 ml solution of either 2 μM CellTracker™ Green (Invitrogen) or 3 µg/ml Hoechst for 40 or 20 min, respectively, at 37°C. Following labelling, cells were centrifuged and mixed with 50% LDEV-free Corning^®^ Matrigel^®^ Basement Membrane Matrix in PBS at a 5:2 ratio of tumour cells:Matrigel^®^. This mixture was injected into the perivitelline space of 2-dpf zebrafish embryos as described (Nicoli and Presta, 2007). The embryos were then washed and placed in E3 media containing 30 mg/l PTU in E3 and incubated at 34°C. FluoSpheres^®^ 1 μm Blue (Invitrogen) grafts, were injected as a 20:1 Fluosphere^®^:Matrigel^®^ mixture.

### Microinjection of clodronate liposomes

PBS or clodronate liposomes (Liposoma) were mixed with a 50% solution of 0.02-0.04 μm fluoresceinated red carboxylated latex beads (Molecular Probes) (as an angiography injection marker to identify successfully injected embryos) in 2% v/v BSA (Sigma-Aldrich) in a 99:1 ratio of liposomes:beads. This injection mixture was sonicated for 5 s and loaded into a borosilicate microinjection needle. At 36 hpf, zebrafish embryos were anaesthetized in a 0.4 mg/ml Tricaine solution and oriented laterally in 2% w/v methylcellulose. A total of 2 nl of the mixture was pipetted into the CCV of the embryo and successful injection of liposomes was confirmed by the appearance of red fluorescence in the bloodstream.

### Nitroreductase-mediated cell ablation

To conduct nitroreductase-mediated macrophage ablation, 36 hpf *mpeg1:NTR:mCherry* embryos were incubated in either 0.5% DMSO (control) or 5 mM metronidazole (Sigma-Aldrich) in E3 solution. To conduct nitroreductase-mediated neutrophil ablation, 36-hpf *mpx:NTR:mCherry* embryos were incubated in 0.75% DMSO (control) or 7.5 mM metronidazole in E3 solution.

### Drug treatment

To test the effects of angiogenesis inhibitor drugs, embryos were incubated in solutions of Tivozanib (AVEO Pharmaceuticals Inc.), SU5402 (Sigma-Aldrich) or DMSO (control) immediately after graft implantation.

### Immunohistochemistry

Embryos were fixed in 4% paraformaldehyde solution in PBS at 4°C overnight, stored at −20°C in methanol and stained according to a previously described fluorescence immunohistochemistry protocol (Turner et al., 2014). The following antibodies were all used at a dilution of 1/500: mouse anti-mCherry (Clontech) with goat anti-mouse Alexa-Fluor^®^-568 secondary (Invitrogen); chicken anti-EGFP (Abcam) with goat anti-chicken Alexa-Fluor^®^-488 secondary (Invitrogen) (Du et al., 2017; Hall et al., 2014).

### Confocal imaging

Live and fixed fish were imaged using a Nikon D-Eclipse C1 confocal scanning microscope by taking optical sections through the graft at 5-µm intervals according to a previously described protocol (Hall et al., 2009).

### Quantitation of graft vascularisation

Graft vascularisation was quantitated using a previously established method (Astin et al., 2014). Using Volocity image analysis software (Improvision^®^, PerkinElmer), the volume of the Hoechst-stained or blue-fluosphere-containing graft was defined by identifying all blue fluorescent objects within a 3D area of interest encompassing the entire graft. A minimum threshold was set for fluorescence intensity (250 units) and object size (100 µm^3^) in order to determine which objects would be defined as part of the graft. The volume of these objects was totalled to give a value for the graft volume. To determine the volume of EGFP-expressing blood vessels associated with the graft, the presence of green fluorescence was identified in a 3D area of interest encompassing all the blue fluorescent objects by using a minimum green fluorescence intensity of 100 units and a minimum volume threshold of 100 µm^3^ to identify green fluorescent objects present in this region. The volume of green fluorescent objects was totalled to give the volume of graft-associated vessels, which was divided by graft volume to give the percentage of graft vascularisation. To measure the graft vascularisation in different regions of the xenograft, we divided each xenograft into three equal sections by volume, labelling the region closest to the CCV as the proximal region, the next region the middle region and the region furthest from the CCV as the distal region (Fig. 5O). Volocity image analysis software (Improvision^®^, PerkinElmer), was used measure the percentage of graft vascularisation in each section as described above.

### Time-lapse imaging

For time-lapse imaging, live fish were imaged using an Olympus FV1000 confocal scanning microscope and the fish were maintained in a Solent incubator at 34°C. The xenografts were imaged every 10 min starting from 8 hpi (MDA-MB-231 xenografts) or 24 hpi (B16-F1 xenografts), and continuing until the experimental endpoint (48 hpi), with optical sections taken at 8-µm intervals.

### Time-lapse analysis

The vessel observed to display the greatest lengthwise growth was chosen and analysed over the period of the movie during which it displayed fastest growth (which usually began at around 24 hpi). During this period (between 5 and 7 h for the MDA-MB-231 and between 3 and 5 h for the B16-F1 xenograft), each frame was analysed by defining a circular region of 10-µm radius centred around the tip of the growing vessel (the angiogenic region) and a control region of the same size located 25 µm away from the angiogenic region but within the graft (Fig. 4H). For each frame analysed, the presence and number of macrophages in each region was counted; for a macrophage to be scored present in a particular region it had to be in the same optical *z*-section(s) as the blood vessel. Tip-cell macrophages were defined as those that associated with the distal tip of a graft blood vessel for at least 40 min. The migration path of the tip-cell macrophage was then tracked for the amount of time that it associated with the tip cell and this analysis was repeated for the same amount of time during the period immediately after it had ceased contact with the tip cell.

### Statistical analysis

All statistical analysis was performed using GraphPad Prism. The Shapiro–Wilk test was used to determine normality of data and, depending on the result, a two-tailed *t*-test (for normally distributed data) or a Mann–Whitney test was used to determine significance. *F*-tests were used to compare variance in order to determine whether to use Welch's correction for the *t*-tests. When comparing multiple sets of data, the one-way ANOVA with Dunnett's multiple comparisons test was used to determine significance of normal data, while the Kruskal–Wallis test was employed for data that were not normally distributed.

## Supplementary Material

Supplementary information
