# Supplementary material for: Macrophages enhance Vegfa-driven angiogenesis in an embryonic zebrafish tumour xenograft model
Source: Dis Model Mech. 2018 Nov 29;11(12):dmm035998. doi: 10.1242/dmm.035998 (PMC6307908; doi:10.1242/dmm.035998)
Supplement: Supplementary information [file dmm-11-035998-s1.pdf]

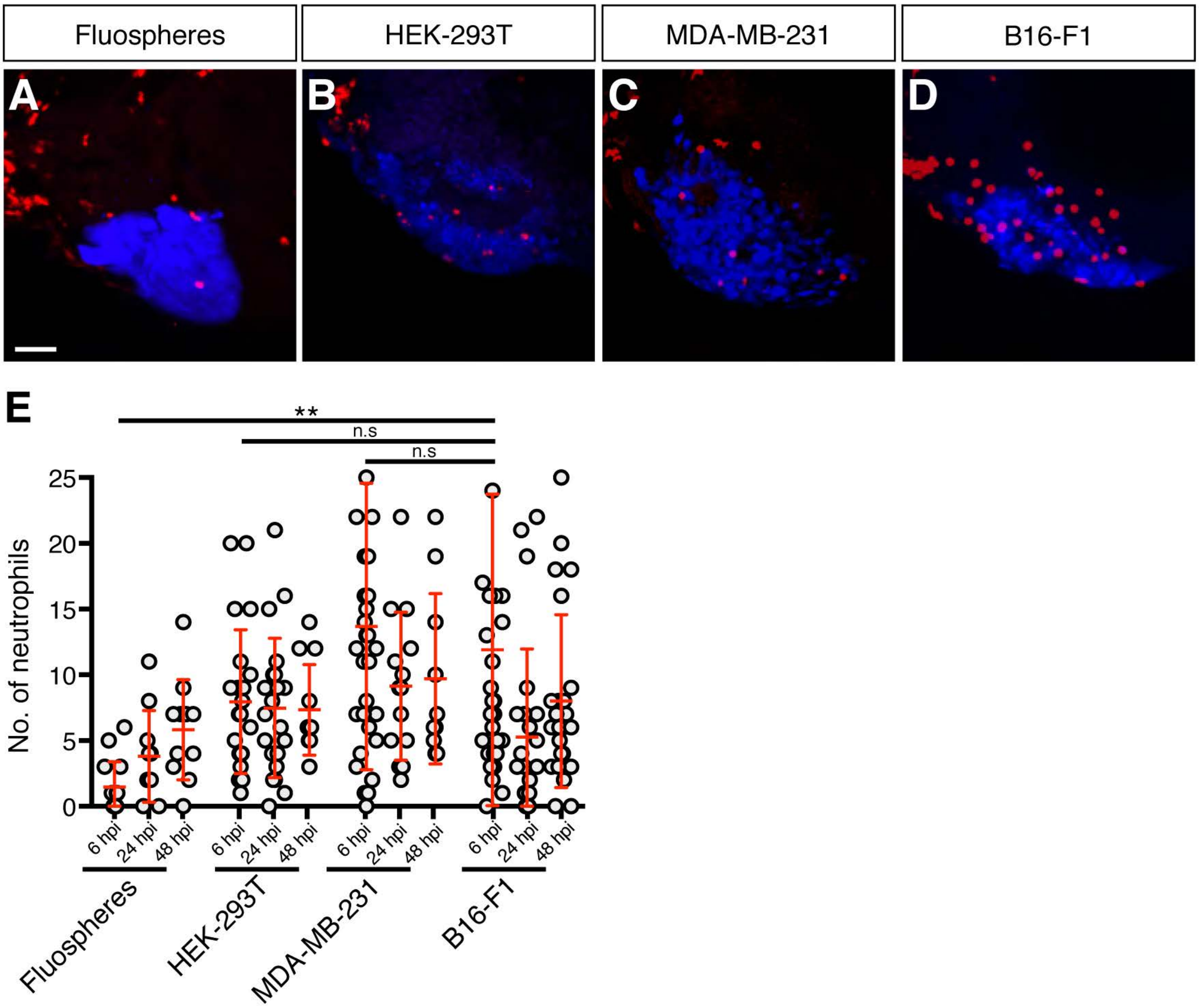

Figure S1. Neutrophils are recruited to grafts.

(A-D) Confocal images of *mpx:mCherry*-expressing neutrophils (red) in zebrafish embryo grafts (blue) at 6 hpi, scale bar = 50 um. **(E)** Quantitation of graft-associated neutrophils at 6, 24 and 48 hpi, n>9. Error bars represent s.d. n.s p>0.05, **p<0.01 by a one-way ANOVA, scale bar = 50 um.

Disease Models & Mechanisms • Supplementary information

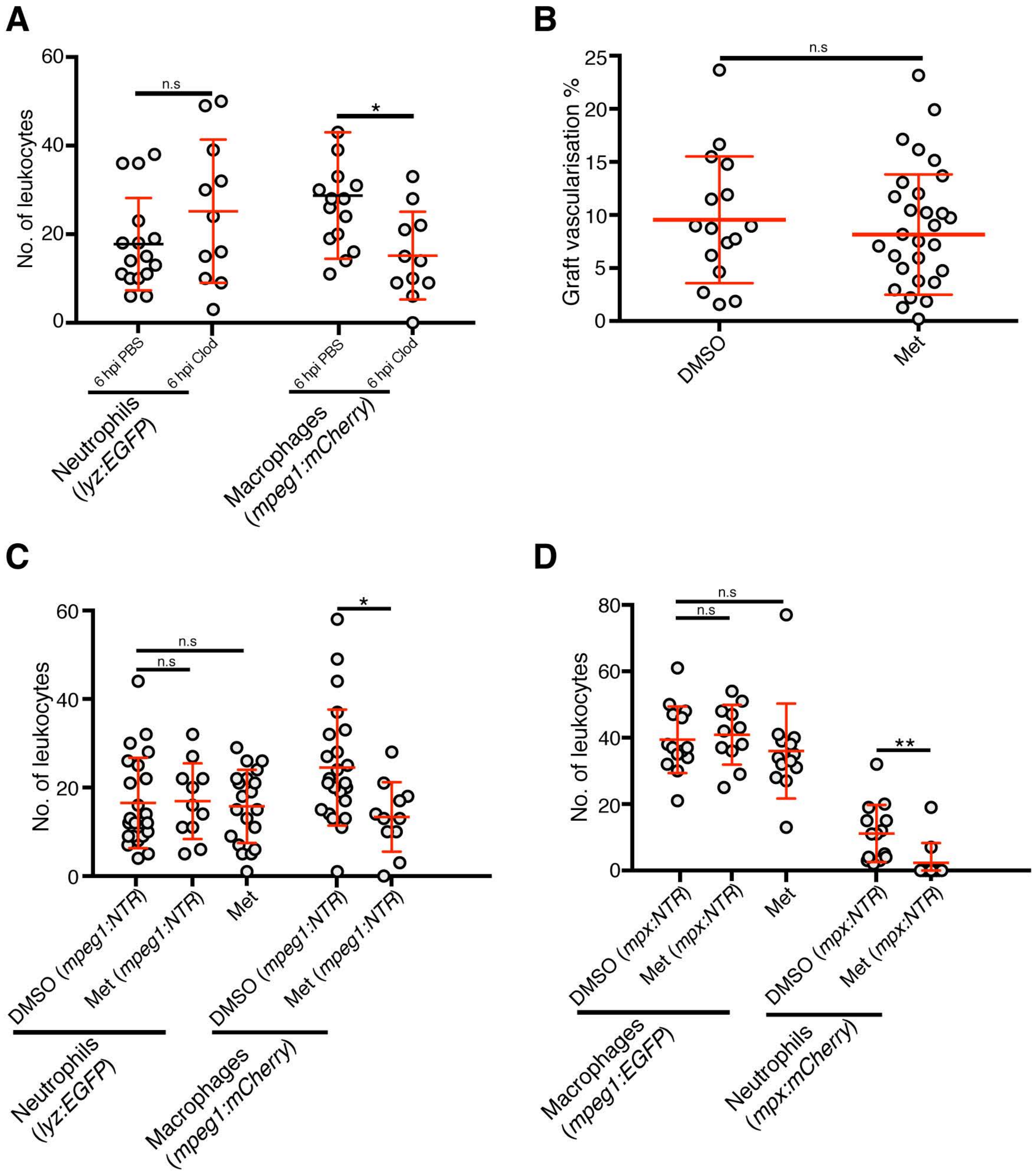
A
No. of leukocytes
60
40
20
0
n.s
*
6 hpi PBS
6 hpi Clod
6 hpi PBS
6 hpi Clod
Neutrophils (lyz:EGFP)
Macrophages (mpeg1:mCherry)
B
Graft vascularisation %
25
20
15
10
5
0
n.s
DMSO
Met
C
No. of leukocytes
n.s
n.s
*
DMSO (mpeg1:NTR)
Met (mpeg1:NTR)
Met
DMSO (mpeg1:NTR)
Met (mpeg1:NTR)
Neutrophils (lyz:EGFP)
Macrophages (mpeg1:mCherry)
D
No. of leukocytes
80
60
40
20
0
n.s
n.s
**
DMSO (mpx:NTR)
Met (mpx:NTR)
Met
DMSO (mpx:NTR)
Met (mpx:NTR)
Macrophages (mpeg1:EGFP)
Neutrophils (mpx:mCherry)

Figure S2. Controls for nitroreductase and clodronate-mediated cell ablations.

(A) Quantitation of MDA-MB-231 xenograft-associated neutrophils and macrophages at 6 hpi in embryos injected with either PBS-containing or clodronate-containing liposomes, n>10. **(B)** Quantitation of graft vascularisation at 2 dpi in MDA-MB-231 xenografts, incubated in 0.75% DMSO or 7.5 mM metronidazole, n>15. **(C)** Quantitation of MDA-MB-231 xenograft-associated neutrophils and macrophages at 6 hpi in either *mpeg1:NTR*-expressing or *NTR*-non-expressing embryos incubated in either 0.75% DMSO or 7.5 mM metronidazole, n>10. **(D)** Quantitation of MDA-MB-231 xenograft-associated macrophages and neutrophils at 6 hpi in *mpx:NTR*-expressing and *NTR* non-expressing embryos incubated in either 0.75% DMSO or 7.5 mM metronidazole, n>10. Error bars represent s.d n.s p>0.05, *p<0.05, **p<0.01 by t-test (A-D), or a one-way ANOVA (C-D).

Disease Models & Mechanisms • Supplementary information

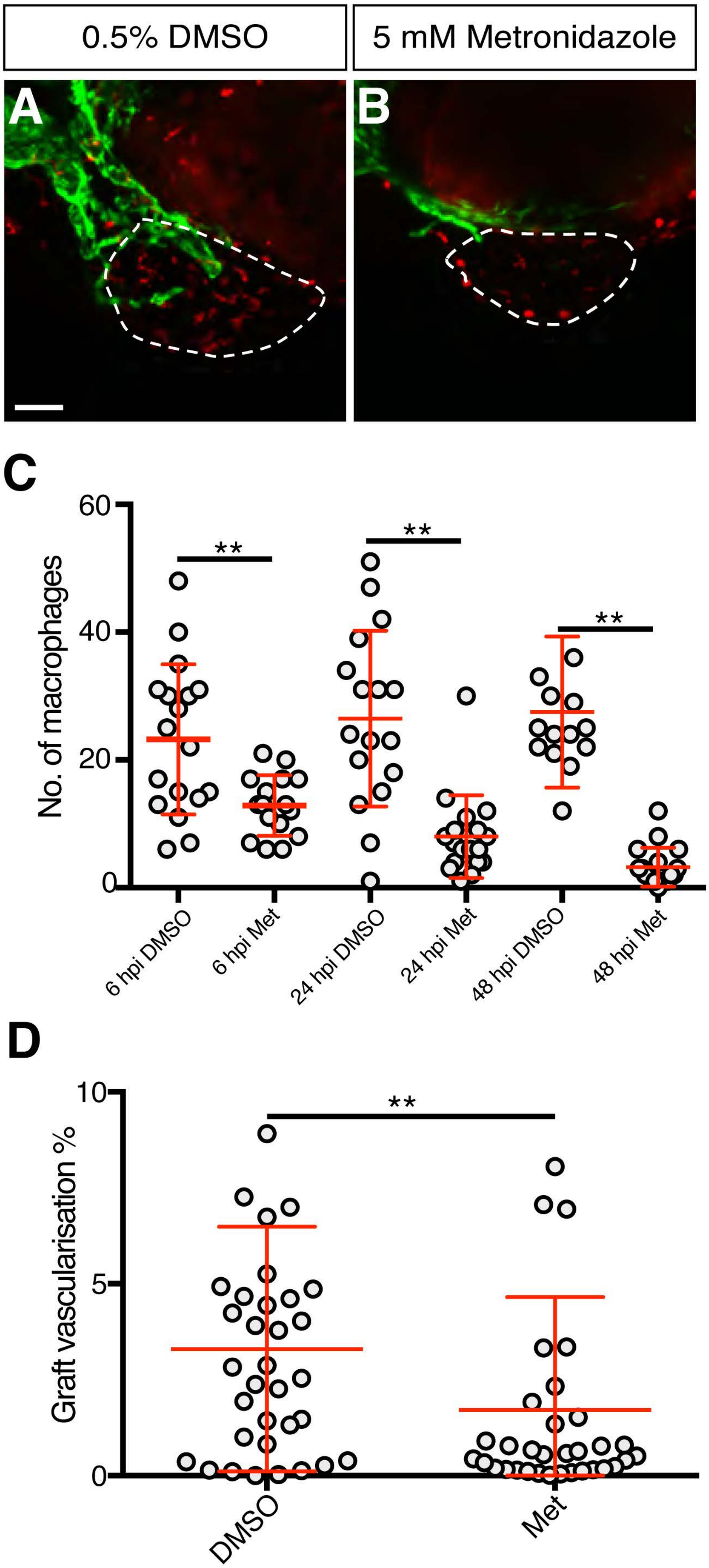

Disease Models & Mechanisms • Supplementary information

Figure S3. Nitroreductase-mediated ablation of macrophages reduced xenograft vascularisation.

(A-B) Confocal images of *kdrl:EGFP*-expressing blood vessels (green) and *mpeg1:mCherry*–expressing macrophages (red) in MDA-MB-231 xenografts (white dashed line) in embryos at 2 dpi incubated in either 0.5% DMSO (A) or 5 mM metronidazole (B). **(C)** Quantitation of xenograft-associated macrophages in embryos incubated in either 0.5% DMSO or 5 mM metronidazole at 6, 24 and 48 hpi, n>15. **(D)** Quantitation of graft vascularisation at 2 dpi in embryos incubated in either 0.5% DMSO or 5 mM metronidazole, n>20. Error bars represent s.d. **p<0.01 by t-test, scale bar = 50 um.

Disease Models & Mechanisms • Supplementary information

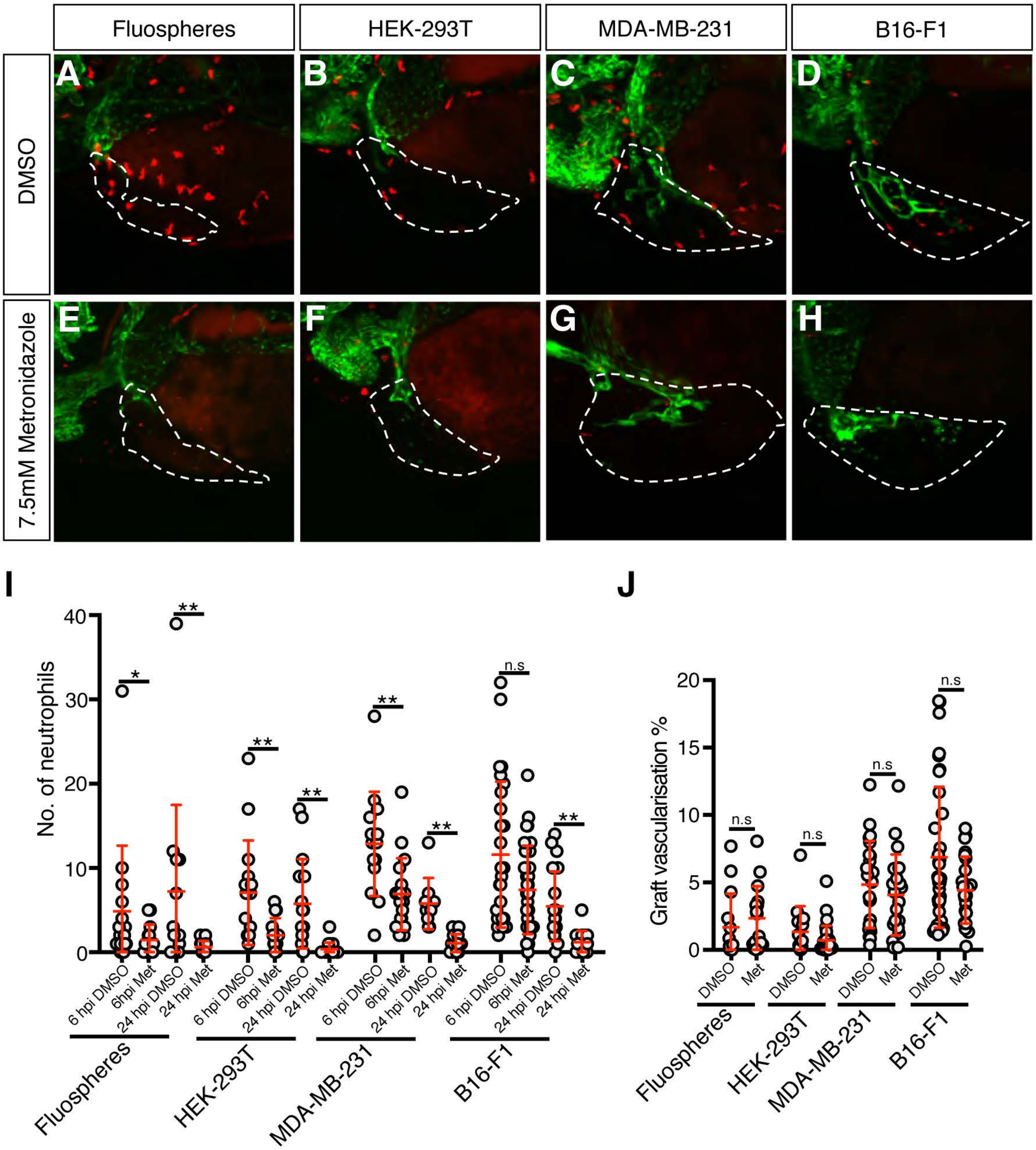
Fluospheres
HEK-293T
MDA-MB-231
B16-F1
DMSO
7.5mM Metronidazole
A
B
C
D
E
F
G
H
I
J
No. of neutrophils
6 hpi DMSO
6hpi Met
24 hpi DMSO
24 hpi Met
n.s
Graft vascularisation %
DMSO
Met

Figure S4. Nitroreductase-mediated ablation of neutrophils does not affect graft vascularisation.

(A-H) Confocal images of *mpx:mCherry*-expressing neutrophils (red) and *kdrl:EGFP*-expressing blood vessels (green) in zebrafish embryo grafts (white dashed line) at 2 dpi, which have been incubated in either 0.75% DMSO (A-D) or 7.5 mM metronidazole (E-H). **(I)** Quantitation of graft-associated neutrophils at 6 and 24 hpi in embryos incubated in either 0.75% DMSO or 7.5 mM metronidazole, n>8. **(J)** Quantitation of graft vascularisation at 2 dpi, in embryos incubated in either 0.75% DMSO or 7.5 mM metronidazole, n>13. Error bars represent s.d. n.s p>0.05, *p<0.05, **p<0.01 by t-test, scale bar = 50 um.

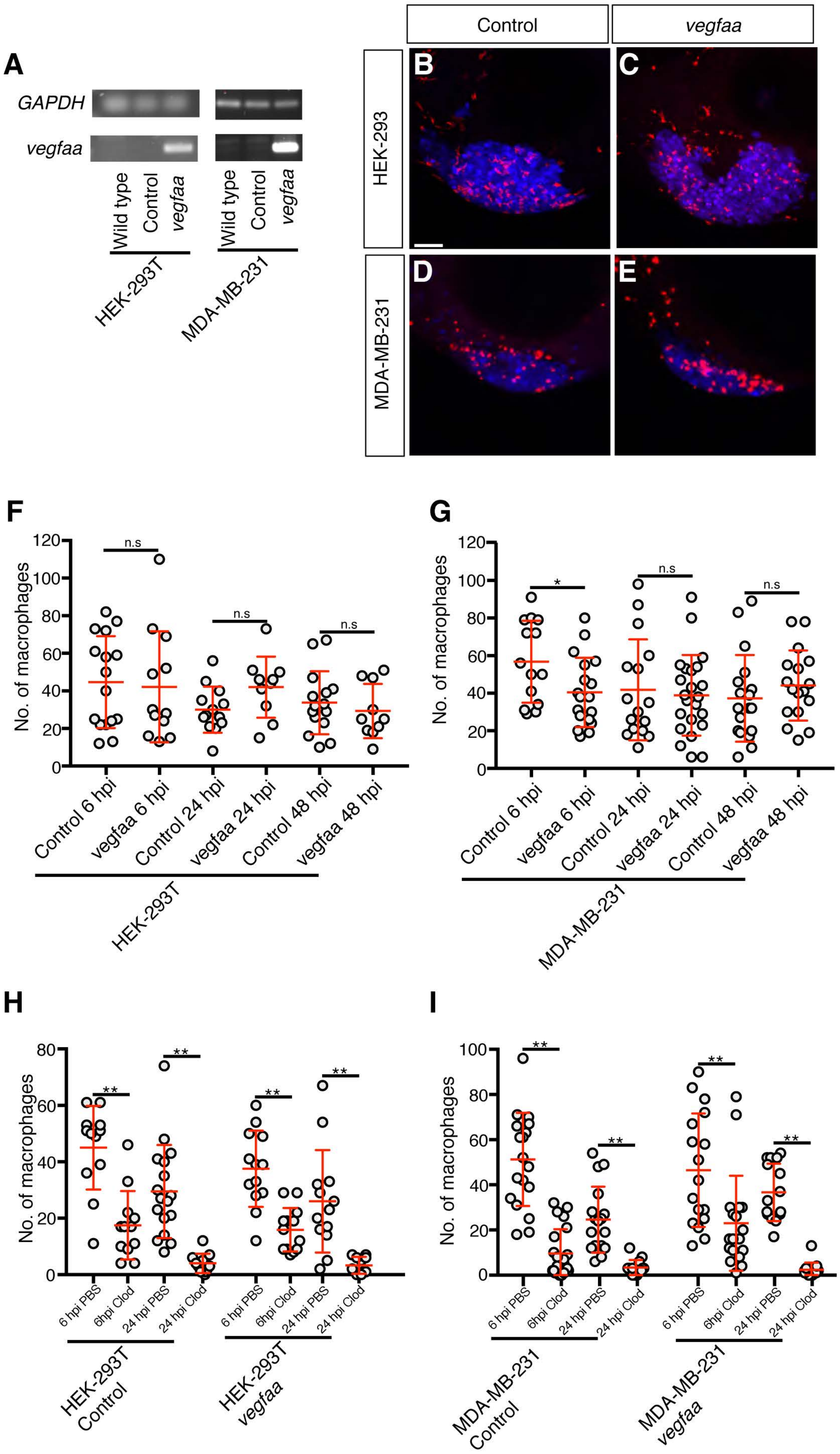

Disease Models & Mechanisms • Supplementary information

Figure S5. Characterisation of *vegfaa*-expressing xenografts.

(A) RT-PCR from HEK-293T and MDA-MB-231 cells transfected with either a control vector or a *vegfaa*-expression vector. (**B-E**) Confocal images taken at 6 hpi of *mpeg1:mCherry*-expressing macrophages (red) in zebrafish embryos implanted with either HEK293T (B-C) or MDA-MB-231 (D-E) xenografts (blue) transfected with either a control expression vector (B,D) or a *vegfaa*-expression vector (C,E). (**F-G**) Quantitation of macrophage recruitment to either HEK-293T (F) or MDA-MB-231 (G) xenografts at 6, 24 and 48 hpi, n>9. (**H-I**) Quantitation of macrophage recruitment to either HEK-293T (H) or MDA-MB-231 (I) xenografts at 6 and 24 hpi in embryos injected with either PBS-containing or clodronate-containing liposomes, n>10. Error bars represent s.d. n.s p>0.05, *p<0.05, **p<0.01 by t-test, scale bar = 50 um.

Disease Models & Mechanisms • Supplementary information

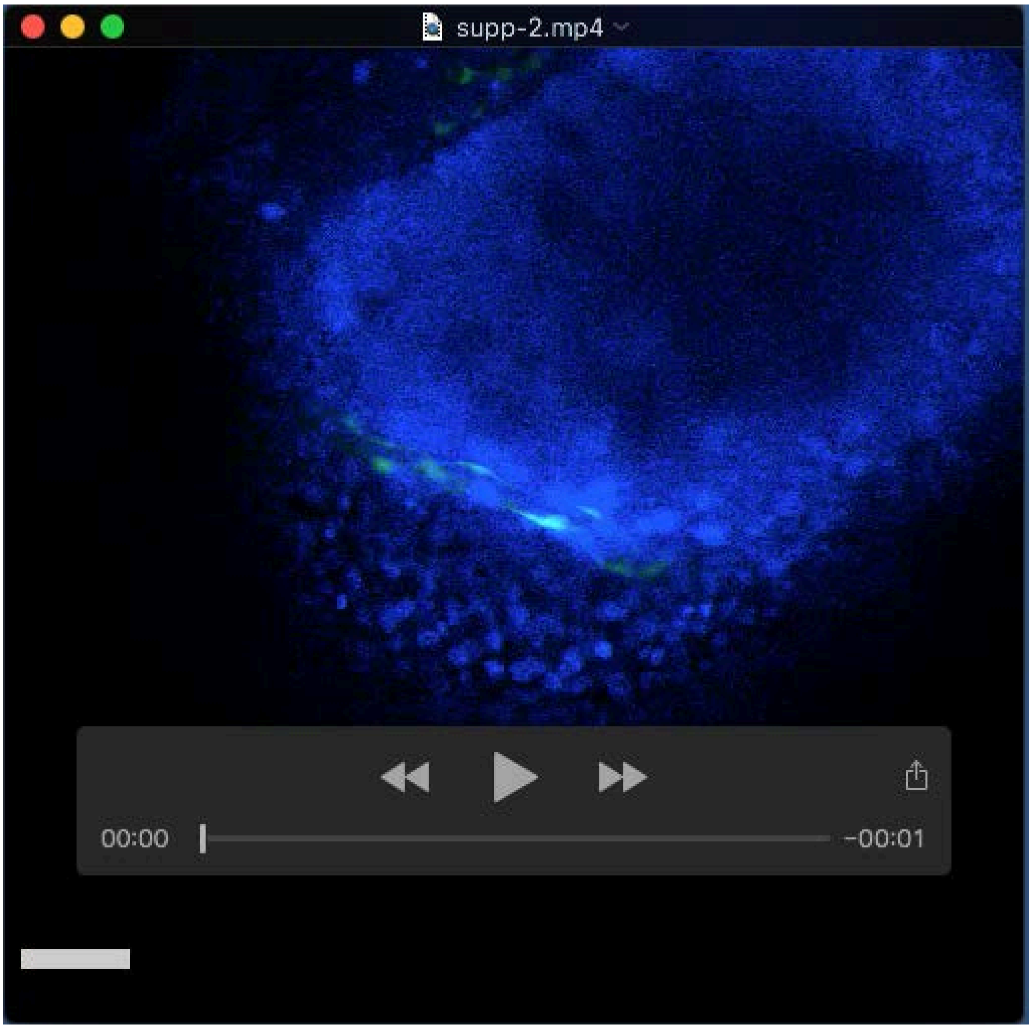

Movie 1. Z-stack of graft vessels in a B16-F1 xenograft in an embryo injected with PBS liposomes.

A movie showing the sections of a confocal z-stack taken through a B16-F1 xenograft (blue) in a *kdrl:EGFP* embryo that had been injected with PBS liposomes. Scale bar = 50 um.

Disease Models & Mechanisms • Supplementary information

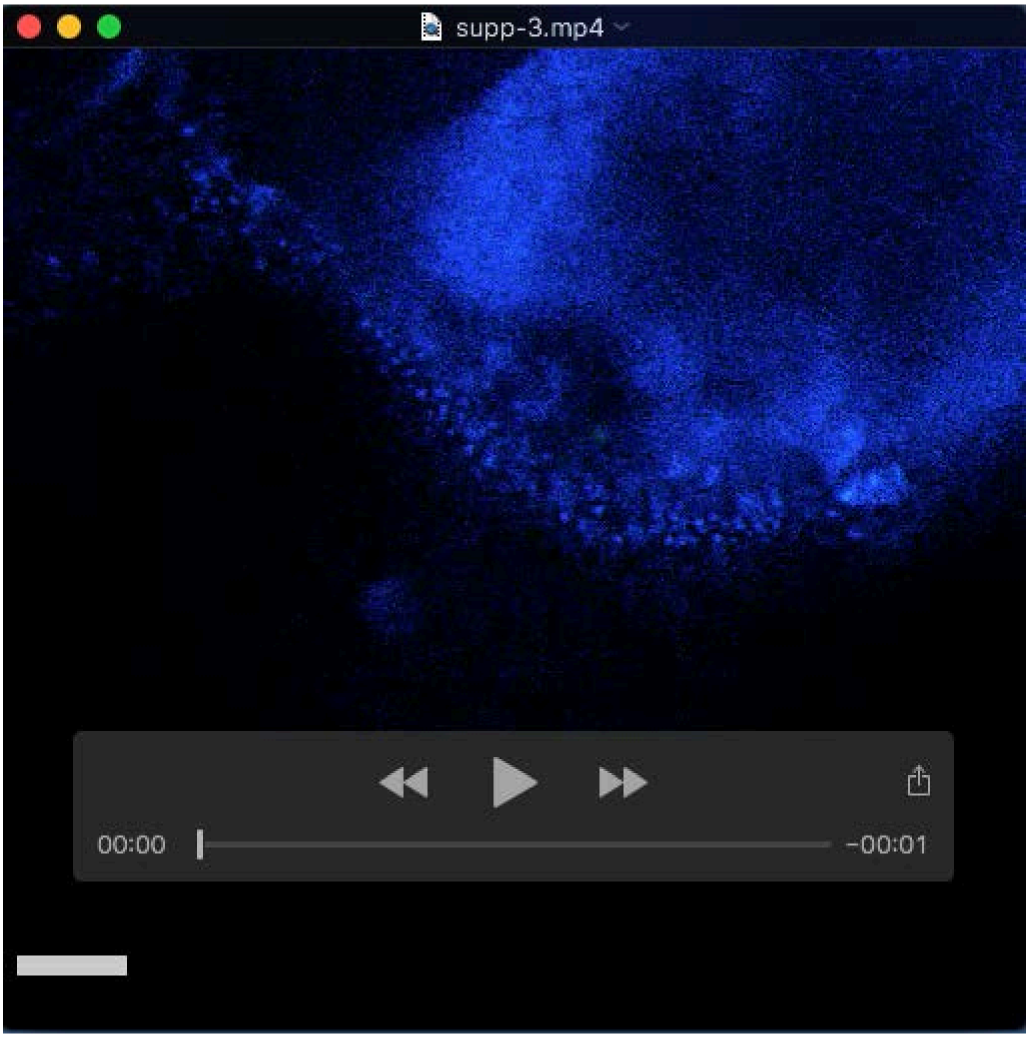

Movie 2. Z-stack of graft vessels in a B16-F1 xenograft in an embryo injected with clodronate liposomes.

A movie showing the sections of a confocal z-stack taken through a B16-F1 xenograft (blue) in a *kdrl:EGFP* embryo that had been injected with clodronate liposomes. Scale bar = 50 um.

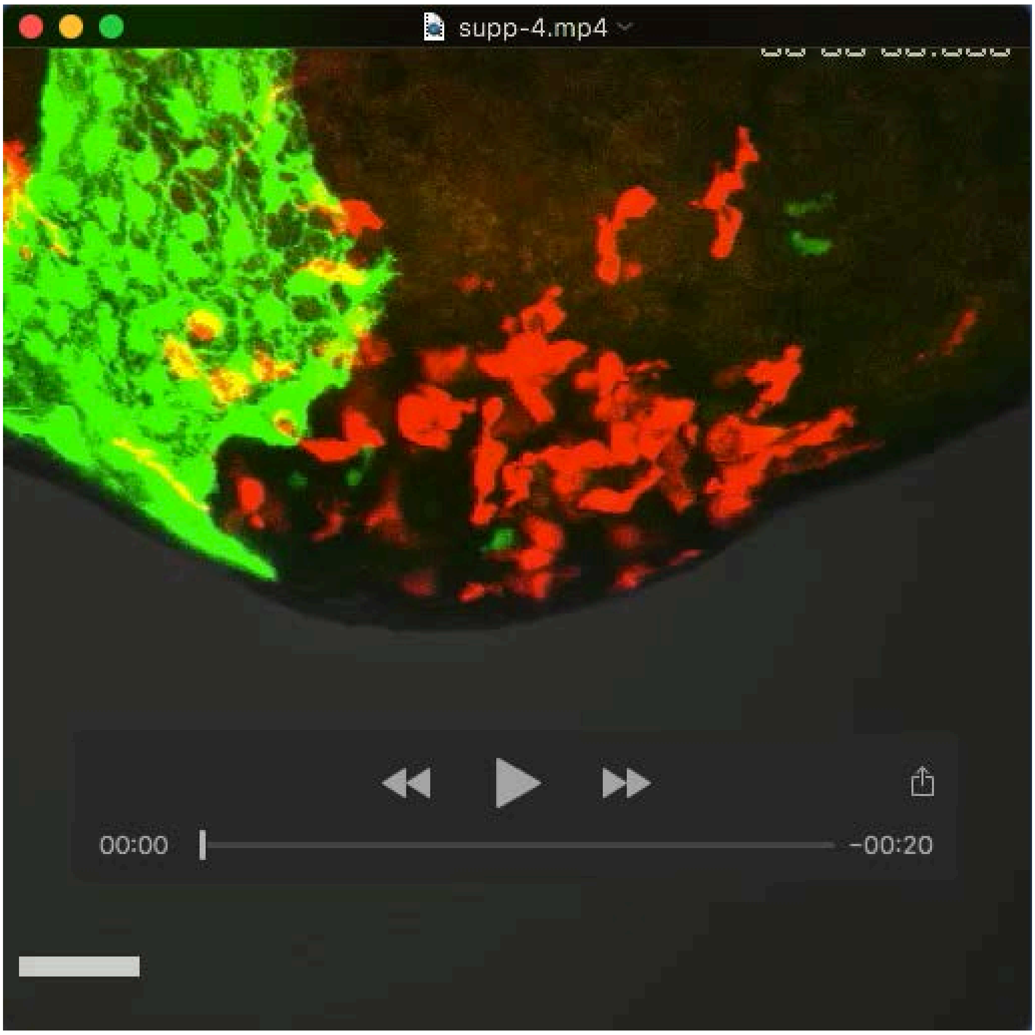

Movie 3. Macrophages interacting with vessels growing in an MDA-MB-231 xenograft.

Confocal time-lapse imaging of an MDA-MB-231 xenograft implanted in an *mpeg1:NTR:mCherry;kdrl:EGFP* embryo from 8.5 hpi to 32 hpi. Time-lapse image z-stacks were collected 10 min apart; movie was made at 7 frames per second. The most dorsal vessel was analysed in Fig. 4 between 21 hpi to 27. 25 hpi. Scale bar = 50 um.

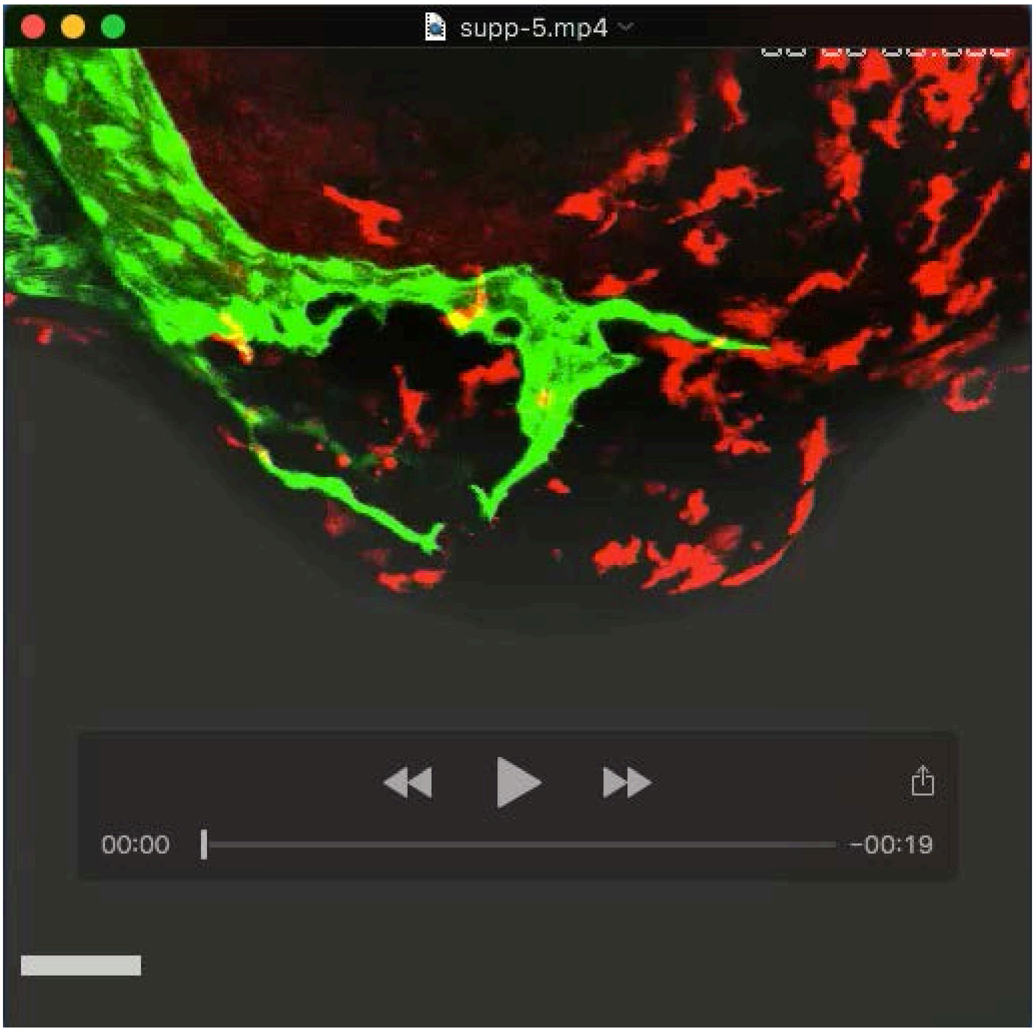

Movie 4. Macrophages interacting with vessels growing in a B16-F1 xenograft. Confocal time-lapse imaging of a B16-F1 xenograft implanted in an *mpeg1:NTR:mCherry;kdrl:EGFP* embryo from 24 hpi to 46.33 hpi. Time-lapse image z-stacks were collected 10 min apart; movie was made at 7 frames per second. The vessel that is most ventral at 27.5 hpi was analysed in Fig. 4 between 27.5 hpi to 32.5 hpi. Scale bar = 50 um.

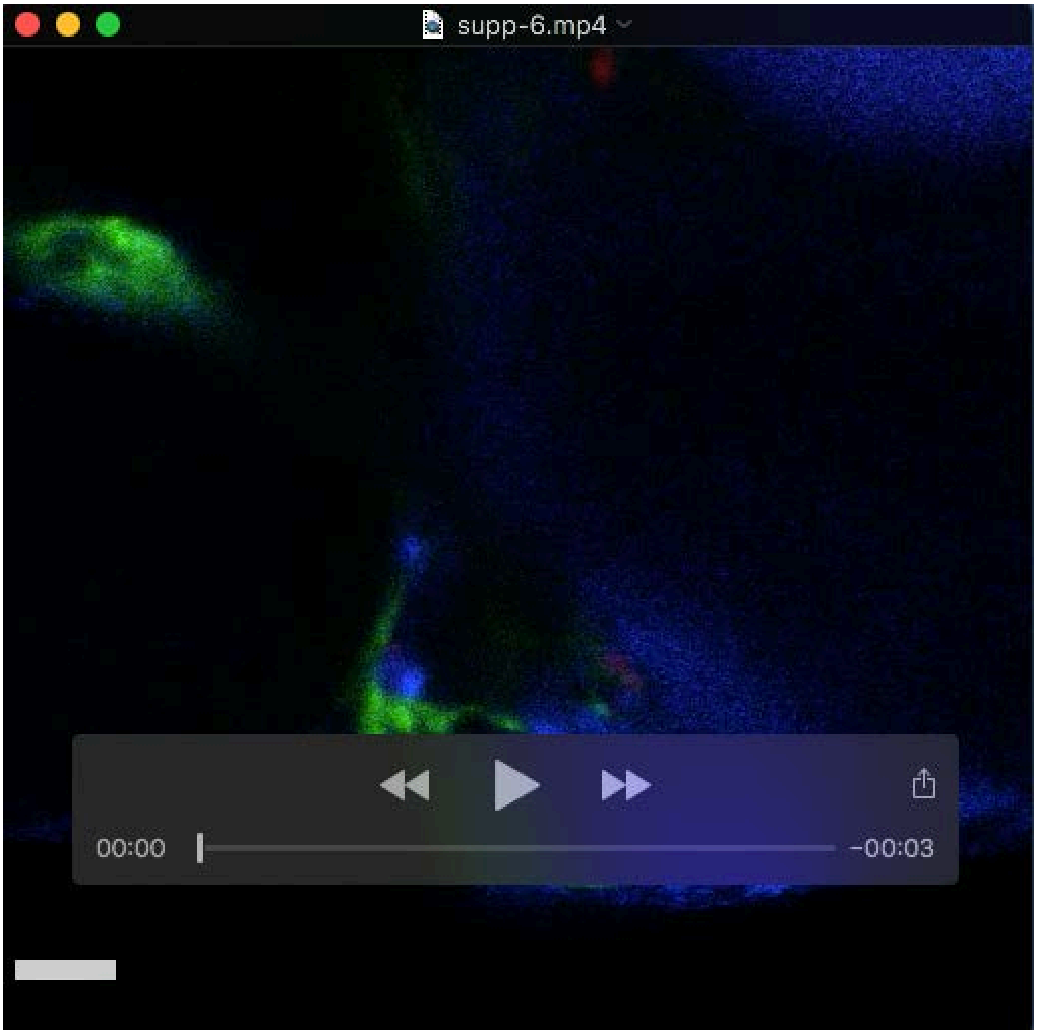

Movie 5. Z-stack of graft vessels in a *vegfaa*-expressing HEK-293T xenograft in an embryo injected with PBS liposomes.

A movie showing the sections of a confocal z-stack taken through a *vegfaa*-expressing HEK-293T xenograft (blue) in a *kdrl:EGFP* embryo that had been injected with PBS liposomes. Scale bar = 50 um.

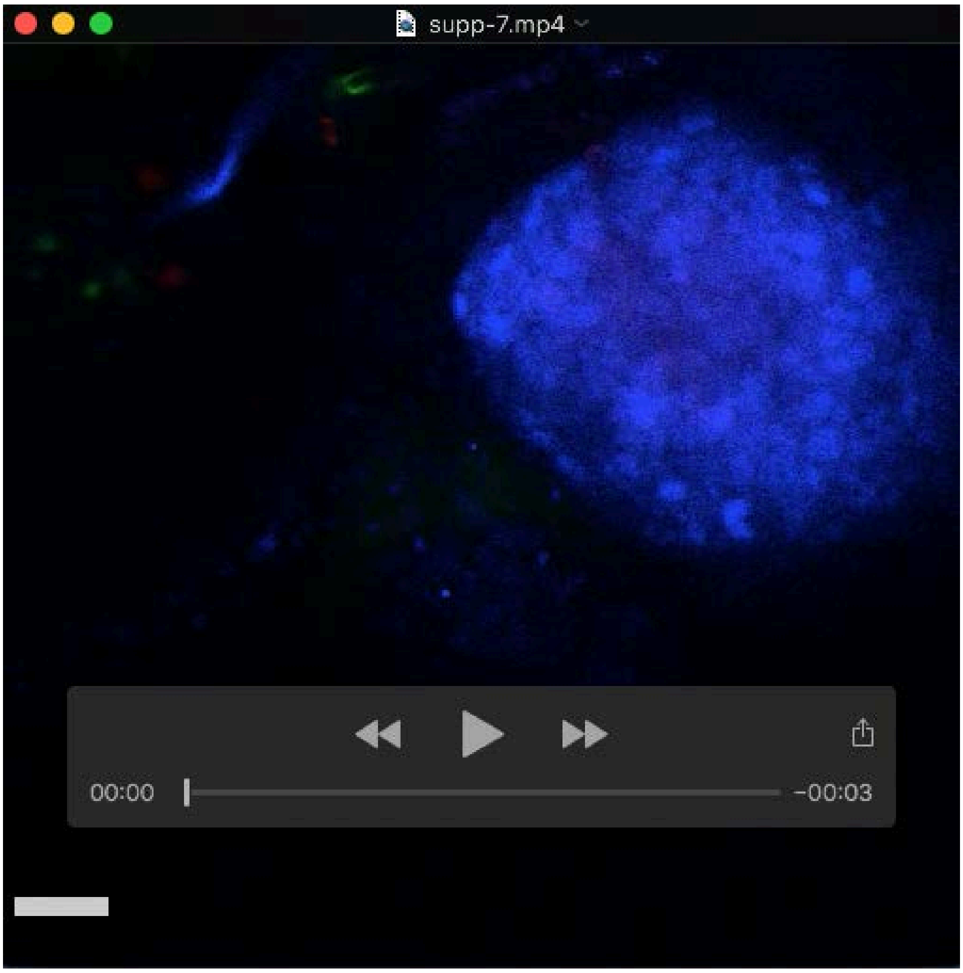

Movie 6. Z-stack of graft vessels in a *vegfaa*-expressing HEK-293T xenograft in an embryo injected with clodronate liposomes.

A movie showing the sections of a confocal z-stack taken through a *vegfaa*-expressing HEK-293T xenograft (blue) in a *kdrl:EGFP* embryo that had been injected with clodronate liposomes. Scale bar = 50 um.

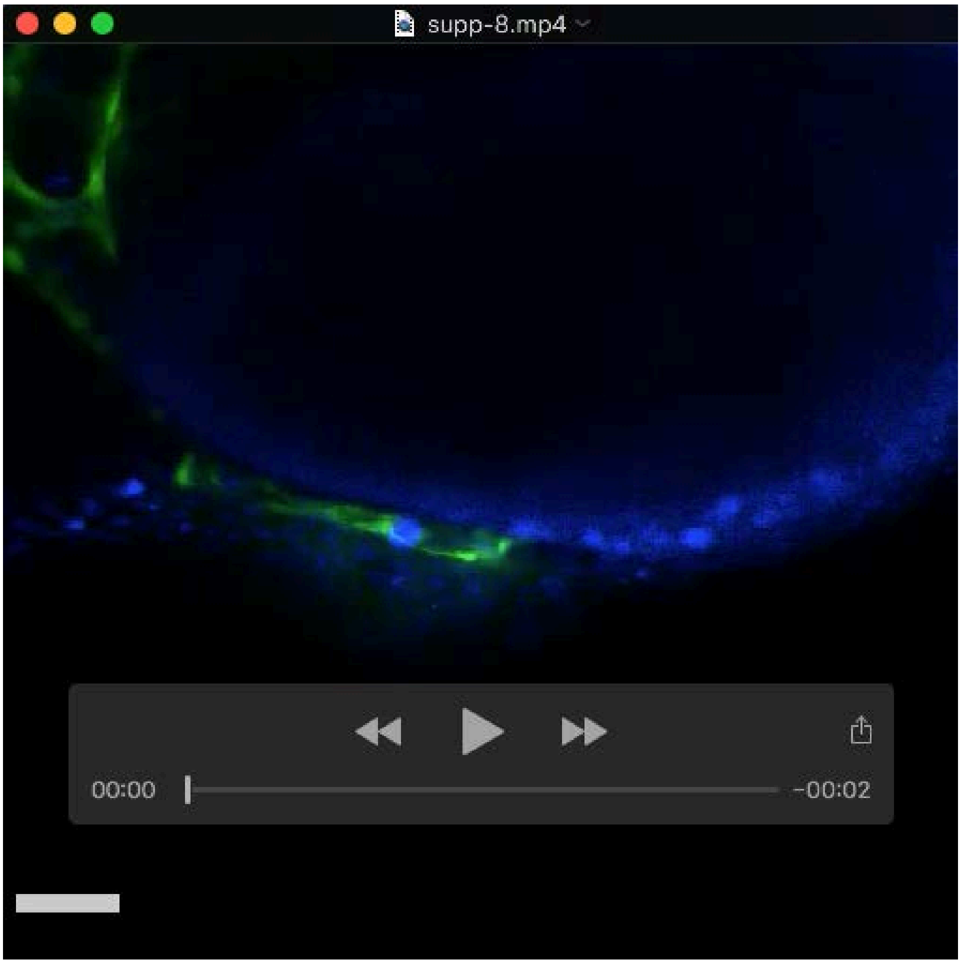

Movie 7. Z-stack of graft vessels in a *vegfaa*-expressing MDA-MB-231 xenograft in an embryo injected with PBS liposomes.

A movie showing the sections of a confocal z-stack taken through a *vegfaa*-expressing MDA-MB-231 xenograft (blue) in a *kdrl:EGFP* embryo that had been injected with PBS liposomes. Scale bar = 50 um.

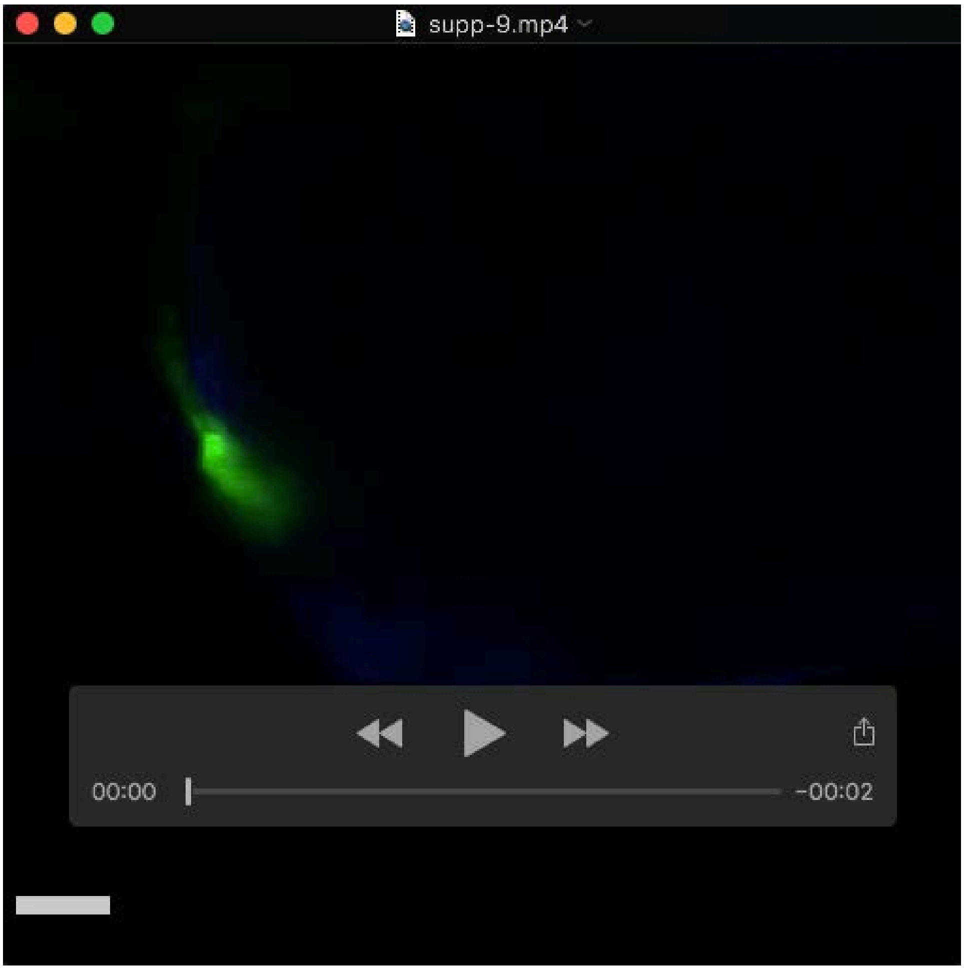

Movie 8. Z-stack of graft vessels in a *vegfaa*-expressing MDA-MB-231 xenograft in an embryo injected with clodronate liposomes.

A movie showing the sections of a confocal z-stack taken through a *vegfaa*-expressing MDA-MB-231 xenograft (blue) in a *kdrl:EGFP* embryo that had been injected with clodronate liposomes. Scale bar = 50 um.

Disease Models & Mechanisms • Supplementary information
